# Supplementary material for: A subset of CB002 xanthine analogs bypass p53-signaling to restore a p53 transcriptome and target an S-phase cell cycle checkpoint in tumors with mutated-p53
Source: eLife. 2021 Jul 29;10:e70429. doi: 10.7554/eLife.70429 (PMC8321552; doi:10.7554/eLife.70429)
Supplement: Source data 1. [file elife-70429-data1.zip › Blots/Hernandez Borrero et al whole western blots final.pptx]

## Slide 1
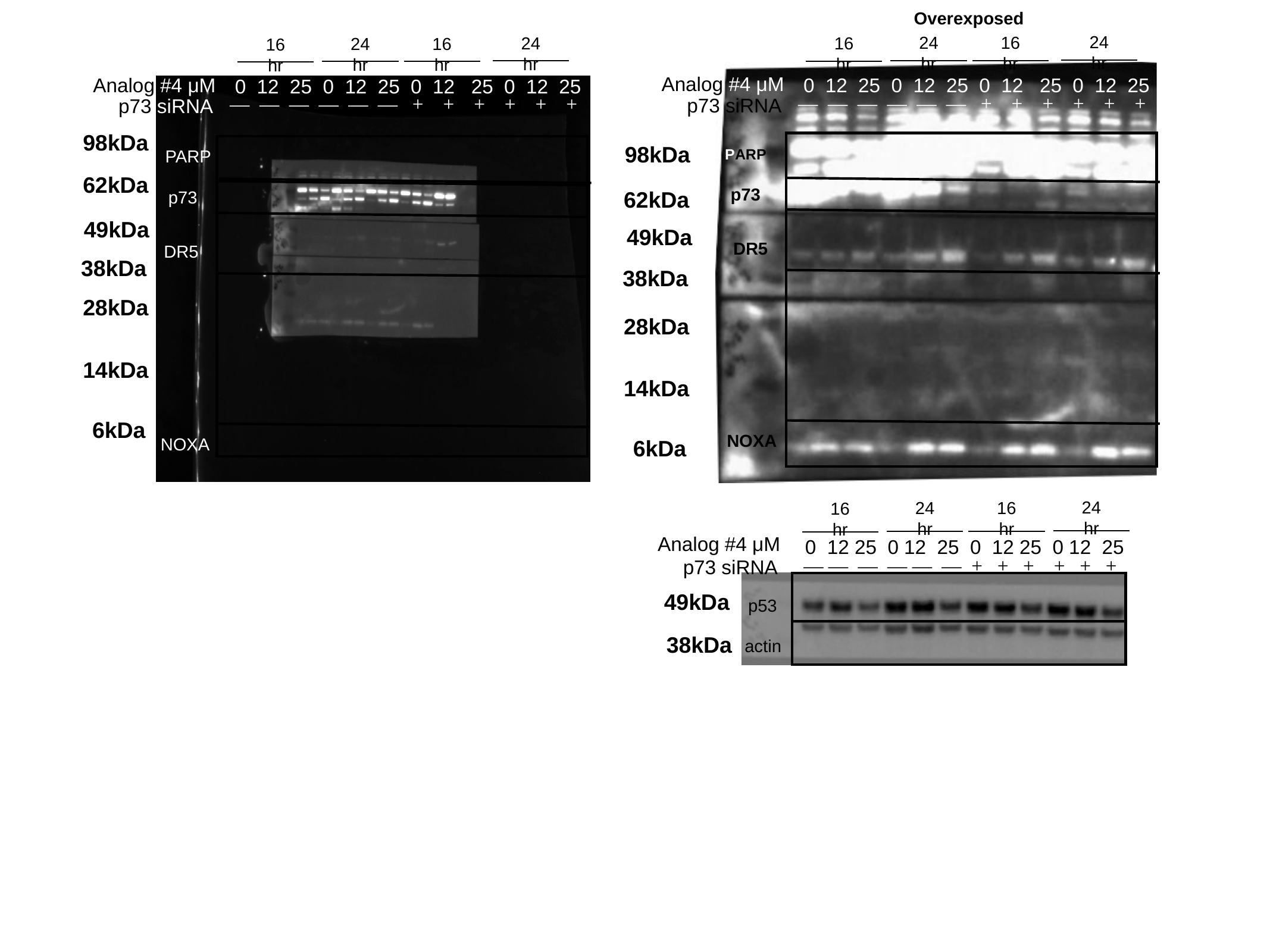

Overexposed
24 hr
24 hr
16 hr
24 hr
16 hr
24 hr
16 hr
16 hr
Analog #4 μM
Analog #4 μM
 0 12 25 0 12 25 0 12 25 0 12 25
 0 12 25 0 12 25 0 12 25 0 12 25
 — — — — — — + + + + + +
 — — — — — — + + + + + +
p73 siRNA
p73 siRNA
98kDa
98kDa
PARP
PARP
62kDa
62kDa
p73
p73
49kDa
49kDa
DR5
DR5
38kDa
38kDa
28kDa
28kDa
14kDa
14kDa
6kDa
NOXA
6kDa
NOXA
24 hr
24 hr
16 hr
16 hr
Analog #4 μM
 0 12 25 0 12 25 0 12 25 0 12 25
 — — — — — — + + + + + +
p73 siRNA
49kDa
p53
38kDa
actin

## Slide 2
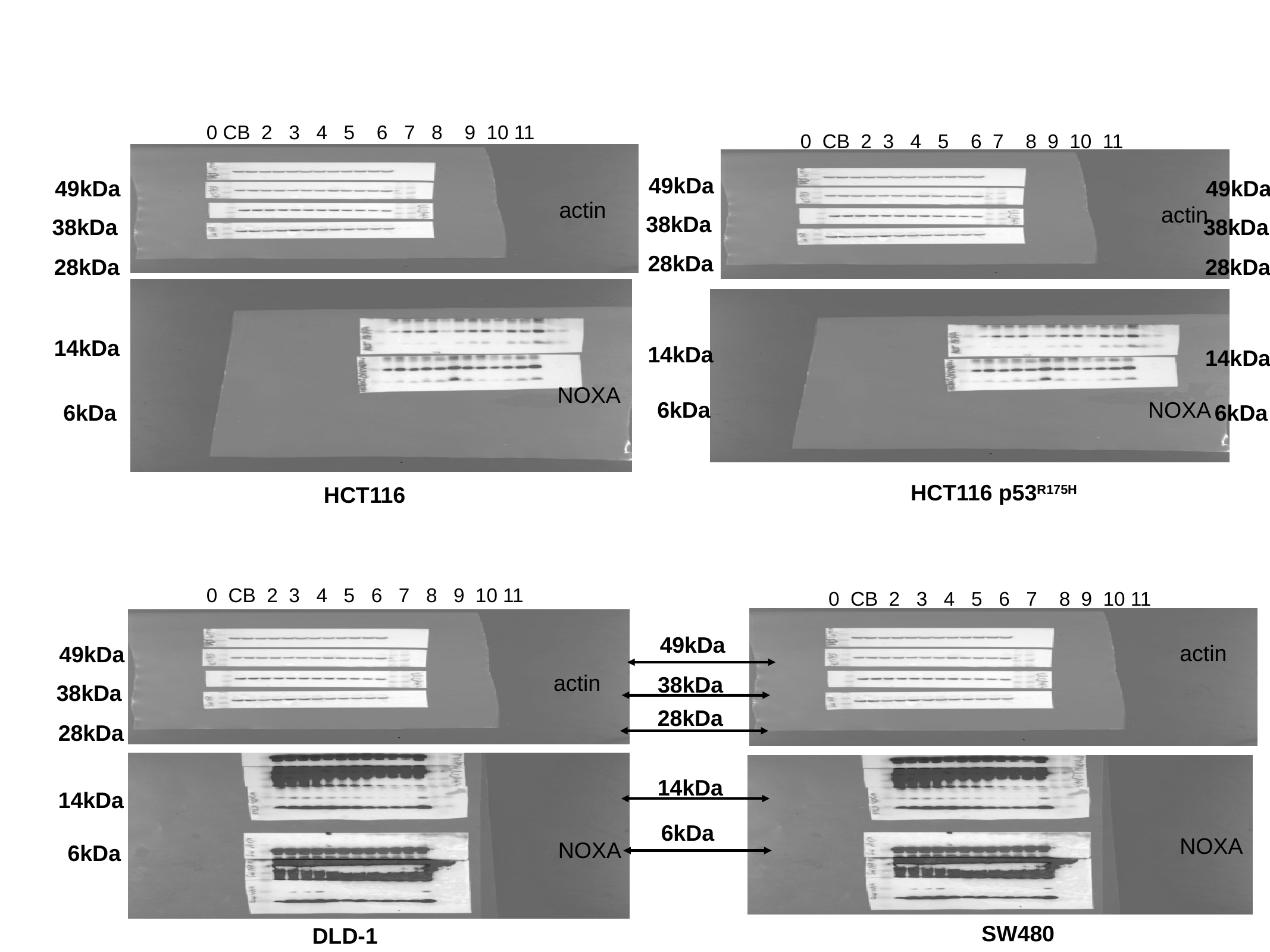

0 CB 2 3 4 5 6 7 8 9 10 11
 0 CB 2 3 4 5 6 7 8 9 10 11
49kDa
49kDa
49kDa
actin
actin
38kDa
38kDa
38kDa
28kDa
28kDa
28kDa
14kDa
14kDa
14kDa
NOXA
6kDa
6kDa
6kDa
NOXA
HCT116 p53R175H
HCT116
 0 CB 2 3 4 5 6 7 8 9 10 11
 0 CB 2 3 4 5 6 7 8 9 10 11
49kDa
49kDa
actin
38kDa
actin
38kDa
28kDa
28kDa
14kDa
14kDa
6kDa
NOXA
6kDa
NOXA
SW480
DLD-1

## Slide 3
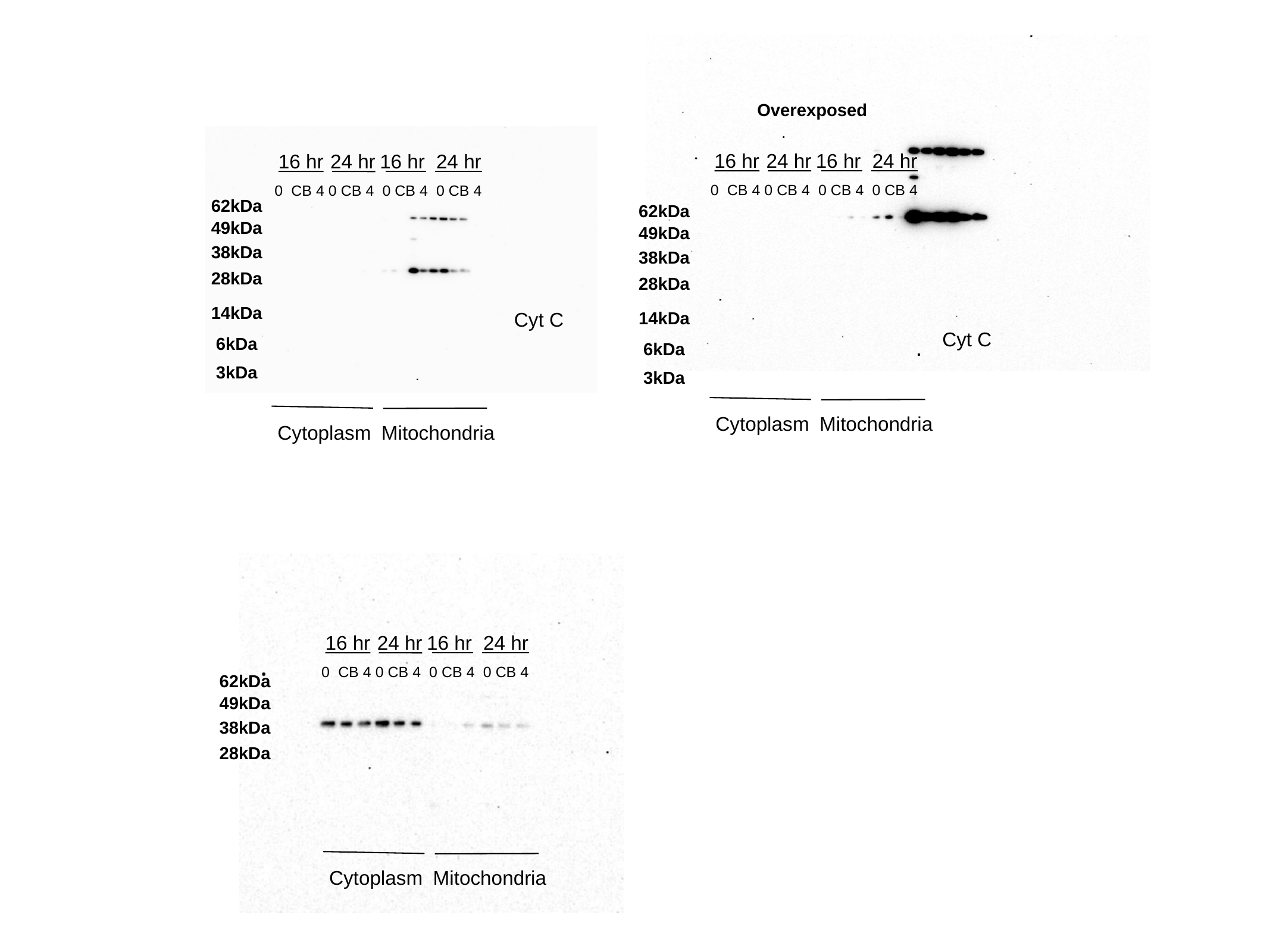

Overexposed
24 hr
16 hr
24 hr
16 hr
24 hr
16 hr
24 hr
16 hr
0 CB 4 0 CB 4 0 CB 4 0 CB 4
0 CB 4 0 CB 4 0 CB 4 0 CB 4
62kDa
62kDa
49kDa
49kDa
38kDa
38kDa
28kDa
28kDa
14kDa
Cyt C
14kDa
Cyt C
6kDa
6kDa
3kDa
3kDa
Cytoplasm
Mitochondria
Cytoplasm
Mitochondria
24 hr
16 hr
24 hr
16 hr
0 CB 4 0 CB 4 0 CB 4 0 CB 4
62kDa
49kDa
38kDa
28kDa
Cytoplasm
Mitochondria

## Slide 4
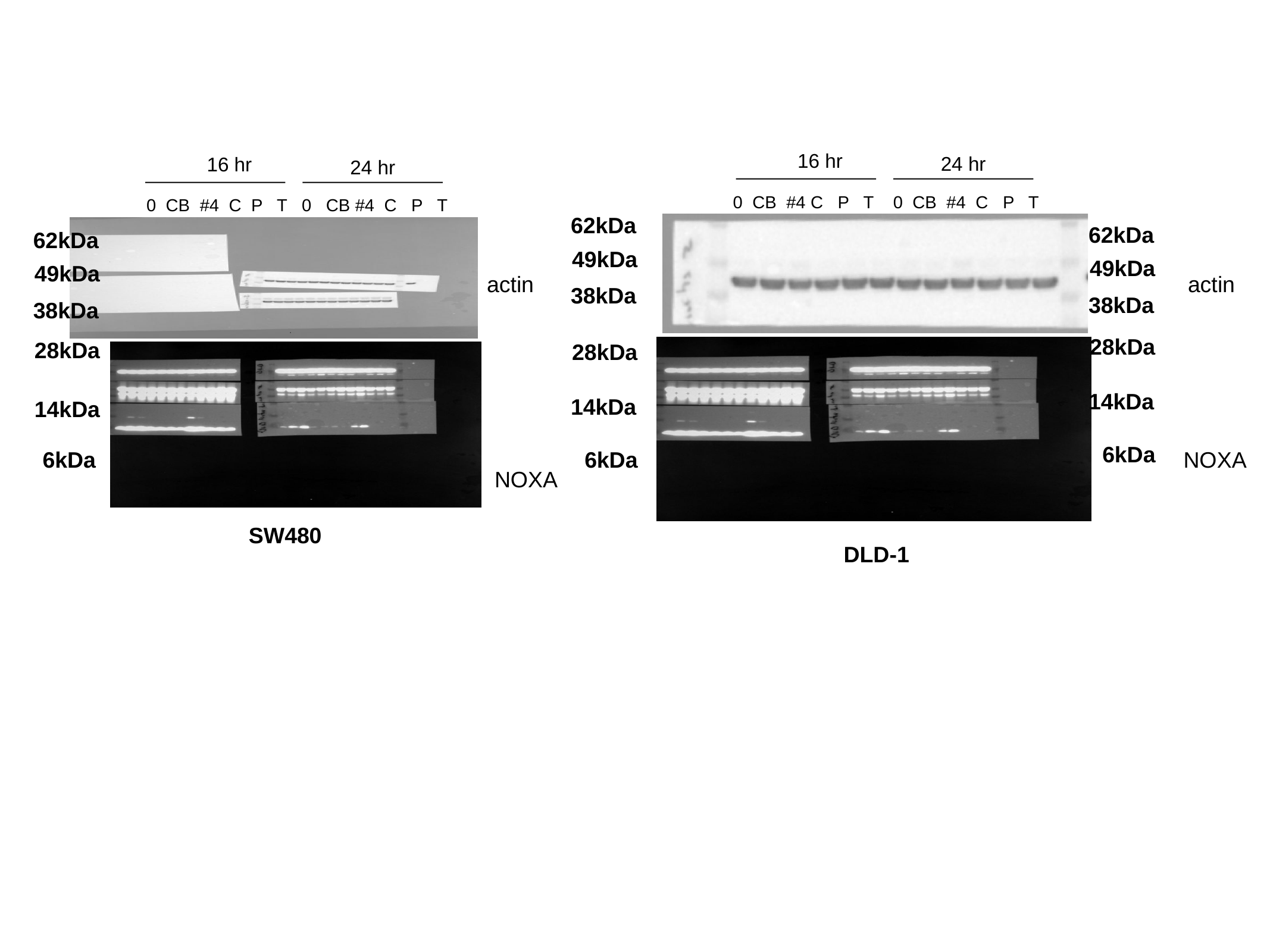

16 hr
24 hr
16 hr
24 hr
 0 CB #4 C P T 0 CB #4 C P T
 0 CB #4 C P T 0 CB #4 C P T
62kDa
62kDa
62kDa
49kDa
49kDa
49kDa
actin
actin
38kDa
38kDa
38kDa
28kDa
28kDa
28kDa
14kDa
14kDa
14kDa
6kDa
6kDa
6kDa
NOXA
NOXA
SW480
DLD-1

## Slide 5
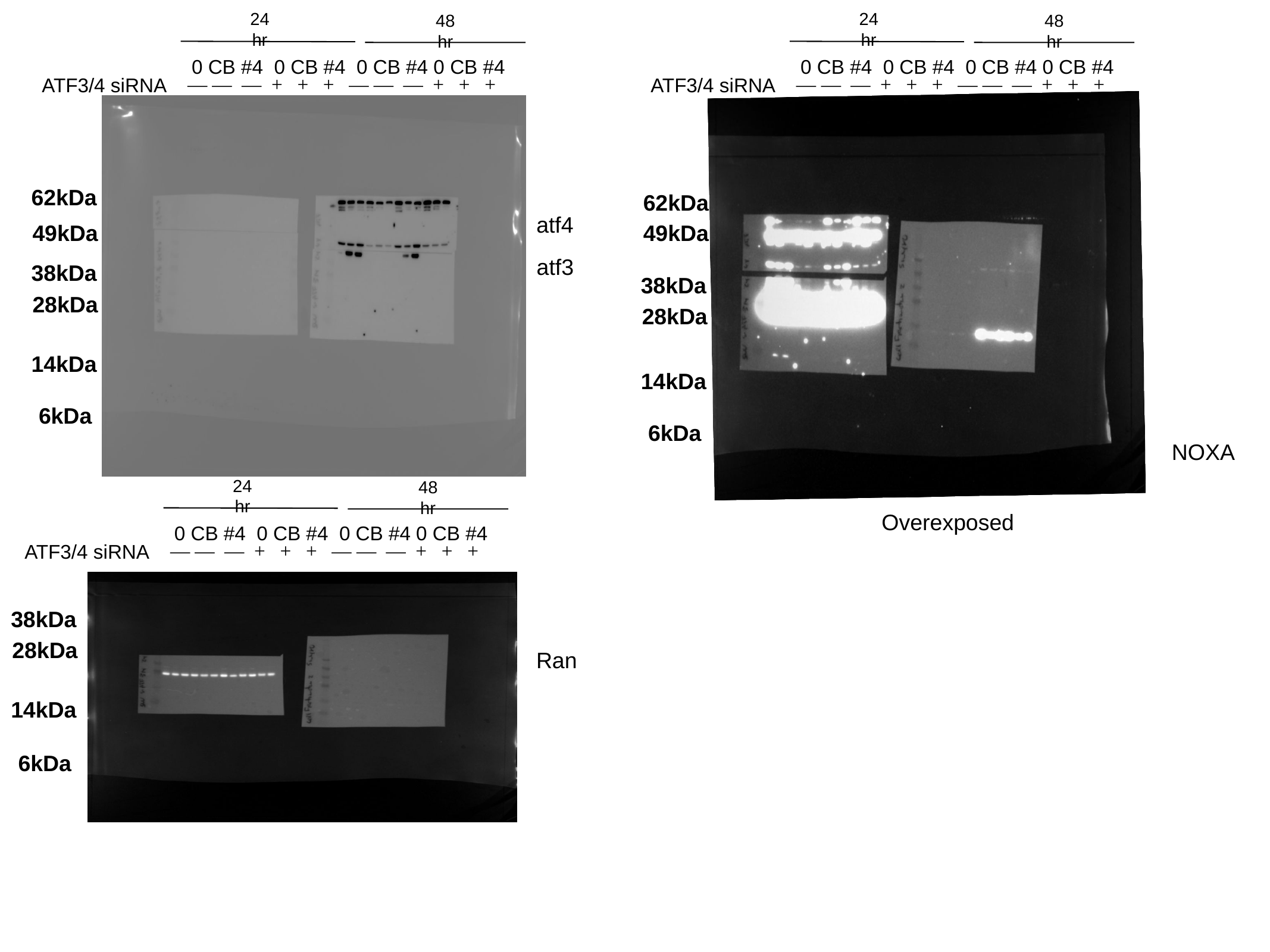

24 hr
24 hr
48 hr
48 hr
 0 CB #4 0 CB #4 0 CB #4 0 CB #4
 0 CB #4 0 CB #4 0 CB #4 0 CB #4
 — — — + + + — — — + + +
 — — — + + + — — — + + +
ATF3/4 siRNA
ATF3/4 siRNA
62kDa
62kDa
49kDa
49kDa
atf4
38kDa
atf3
38kDa
28kDa
28kDa
14kDa
14kDa
6kDa
6kDa
NOXA
24 hr
48 hr
Overexposed
 0 CB #4 0 CB #4 0 CB #4 0 CB #4
 — — — + + + — — — + + +
ATF3/4 siRNA
38kDa
28kDa
Ran
14kDa
6kDa

## Slide 6
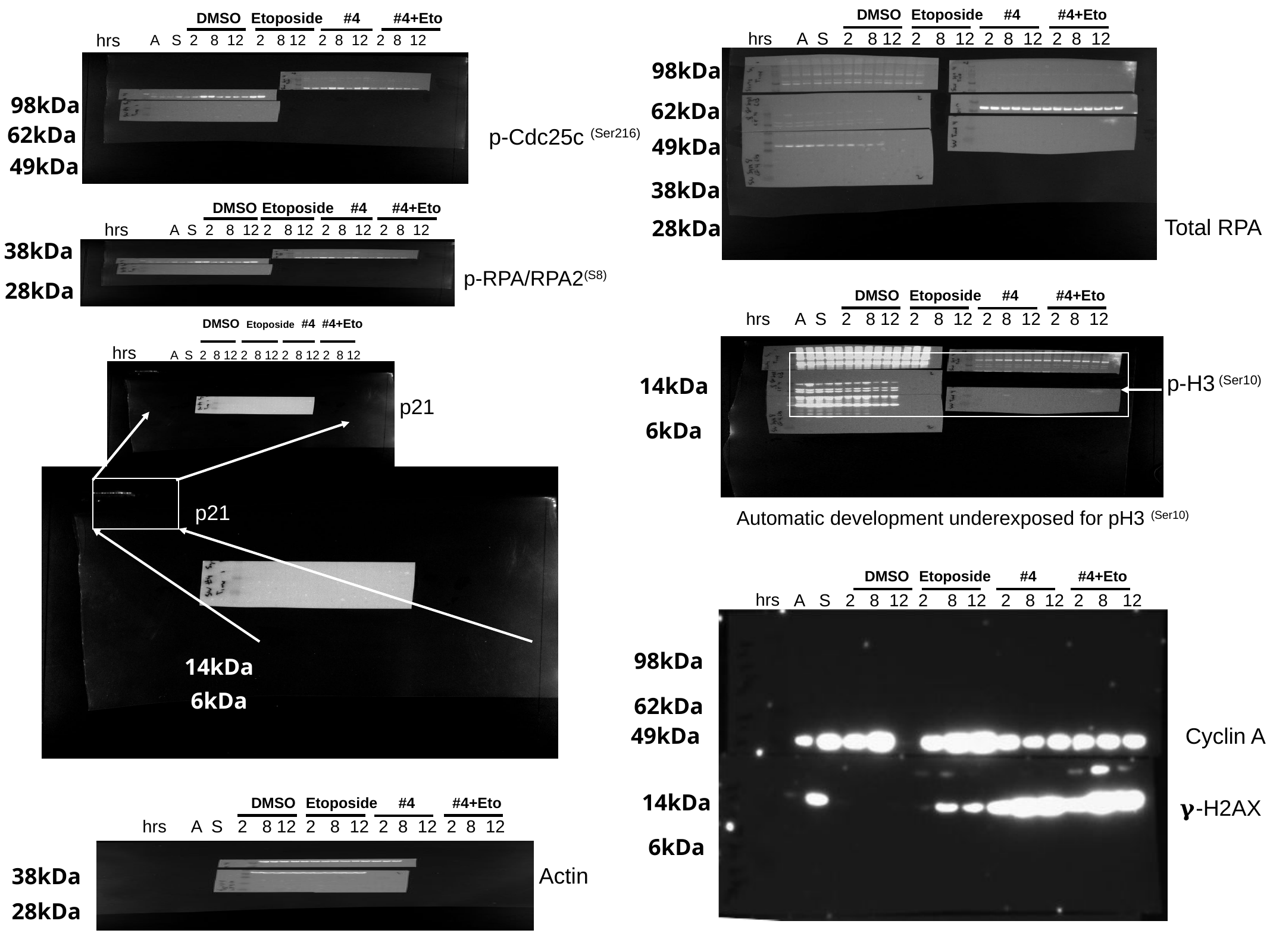

DMSO Etoposide #4 #4+Eto
 DMSO Etoposide #4 #4+Eto
hrs
A S 2 8 12 2 8 12 2 8 12 2 8 12
hrs
 A S 2 8 12 2 8 12 2 8 12 2 8 12
98kDa
98kDa
62kDa
62kDa
p-Cdc25c (Ser216)
49kDa
49kDa
38kDa
 DMSO Etoposide #4 #4+Eto
Total RPA
28kDa
hrs
 A S 2 8 12 2 8 12 2 8 12 2 8 12
38kDa
p-RPA/RPA2(S8)
28kDa
 DMSO Etoposide #4 #4+Eto
hrs
A S 2 8 12 2 8 12 2 8 12 2 8 12
 DMSO Etoposide #4 #4+Eto
hrs
A S 2 8 12 2 8 12 2 8 12 2 8 12
p-H3 (Ser10)
14kDa
p21
6kDa
p21
Automatic development underexposed for pH3 (Ser10)
 DMSO Etoposide #4 #4+Eto
hrs
A S 2 8 12 2 8 12 2 8 12 2 8 12
98kDa
14kDa
6kDa
62kDa
Cyclin A
49kDa
14kDa
 DMSO Etoposide #4 #4+Eto
𝛄-H2AX
hrs
A S 2 8 12 2 8 12 2 8 12 2 8 12
6kDa
Actin
38kDa
28kDa

## Slide 7
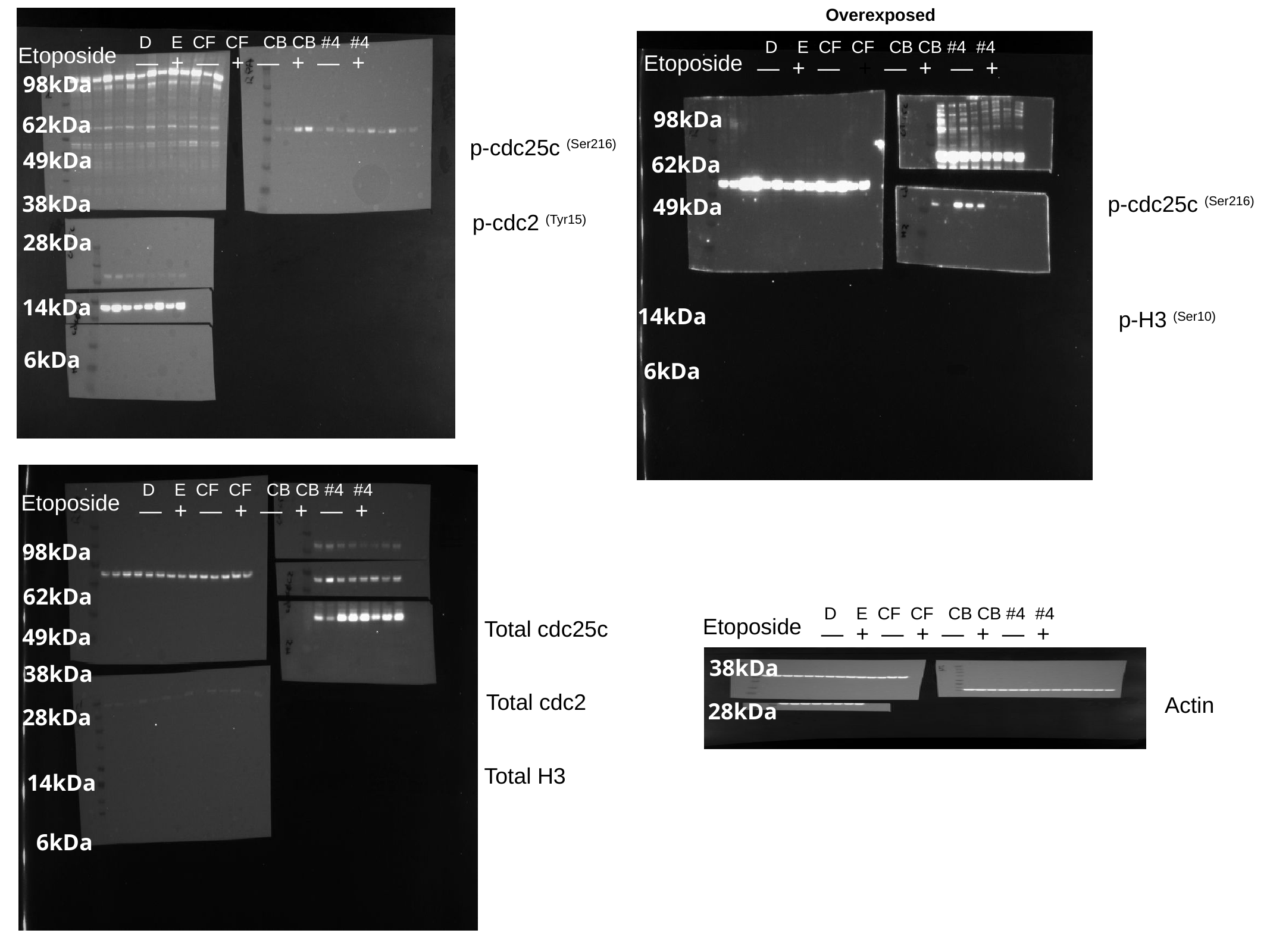

Overexposed
 D E CF CF CB CB #4 #4
 D E CF CF CB CB #4 #4
Etoposide
 — + — + — + — +
Etoposide
 — + — + — + — +
98kDa
98kDa
62kDa
p-cdc25c (Ser216)
49kDa
62kDa
p-cdc25c (Ser216)
38kDa
49kDa
p-cdc2 (Tyr15)
28kDa
14kDa
14kDa
p-H3 (Ser10)
6kDa
6kDa
 D E CF CF CB CB #4 #4
Etoposide
 — + — + — + — +
98kDa
62kDa
 D E CF CF CB CB #4 #4
Etoposide
Total cdc25c
 — + — + — + — +
49kDa
38kDa
38kDa
Total cdc2
Actin
28kDa
28kDa
Total H3
14kDa
6kDa

## Slide 8
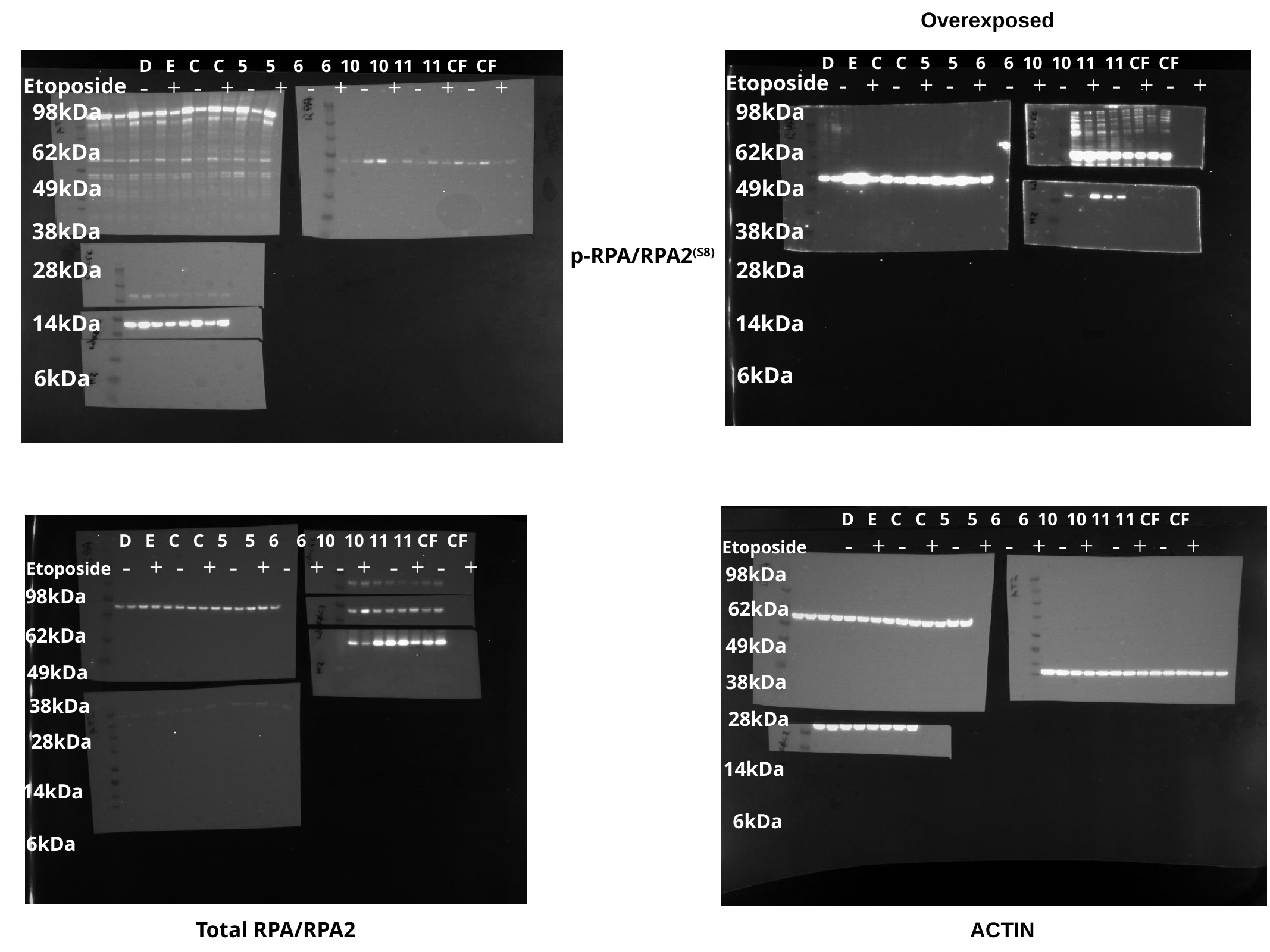

Overexposed
D E C C 5 5 6 6 10 10 11 11 CF CF
D E C C 5 5 6 6 10 10 11 11 CF CF
Etoposide
 - + - + - + - + - + - + - +
Etoposide
 - + - + - + - + - + - + - +
98kDa
98kDa
62kDa
62kDa
49kDa
49kDa
38kDa
38kDa
p-RPA/RPA2(S8)
28kDa
28kDa
14kDa
14kDa
6kDa
6kDa
D E C C 5 5 6 6 10 10 11 11 CF CF
D E C C 5 5 6 6 10 10 11 11 CF CF
 - + - + - + - + - + - + - +
Etoposide
 - + - + - + - + - + - + - +
Etoposide
98kDa
98kDa
62kDa
62kDa
49kDa
49kDa
38kDa
38kDa
28kDa
28kDa
14kDa
14kDa
6kDa
6kDa
Total RPA/RPA2
ACTIN

## Slide 9
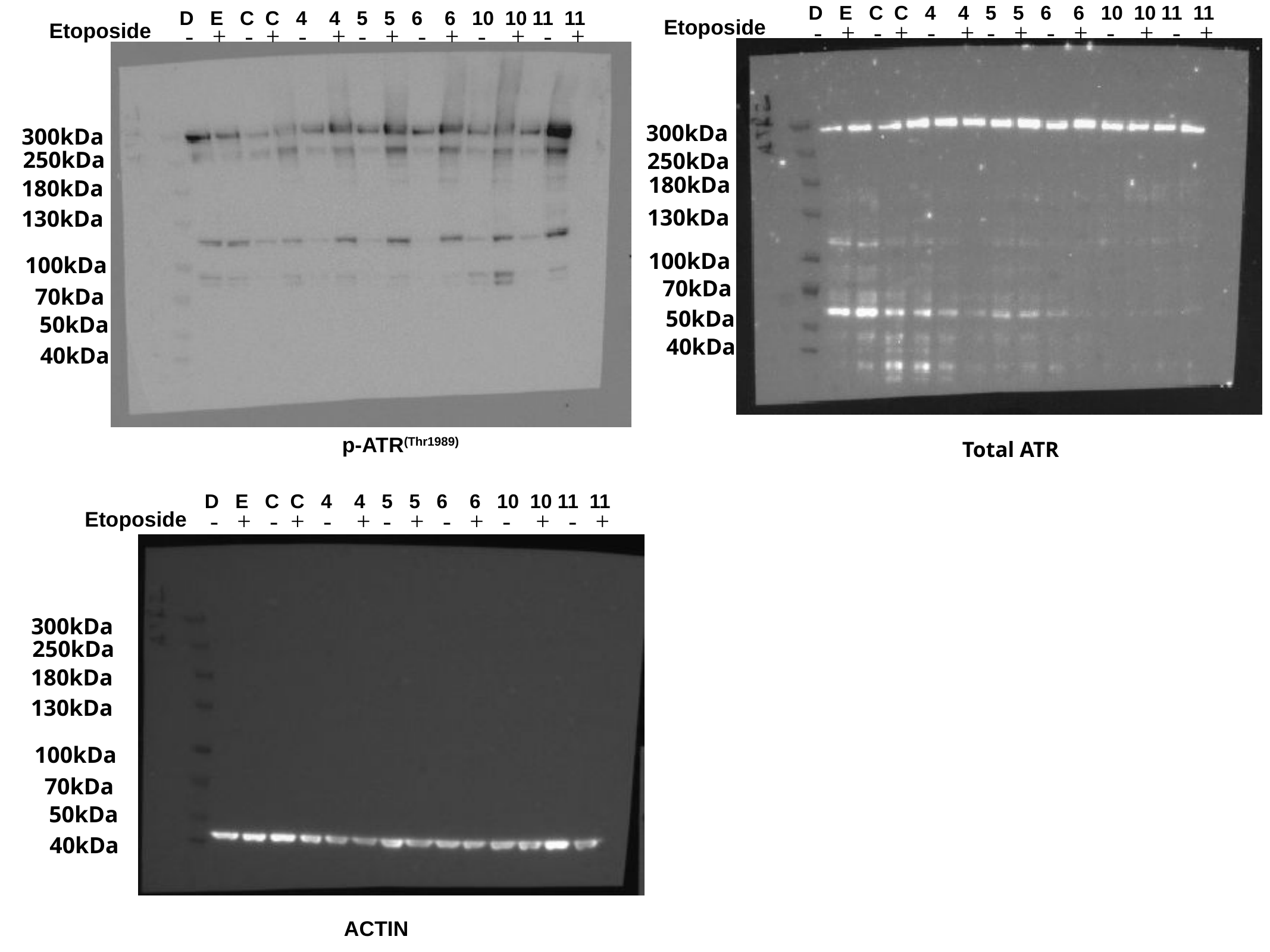

D E C C 4 4 5 5 6 6 10 10 11 11
D E C C 4 4 5 5 6 6 10 10 11 11
Etoposide
Etoposide
 - + - + - + - + - + - + - +
 - + - + - + - + - + - + - +
300kDa
300kDa
250kDa
250kDa
180kDa
180kDa
130kDa
130kDa
100kDa
100kDa
70kDa
70kDa
50kDa
50kDa
40kDa
40kDa
p-ATR(Thr1989)
Total ATR
D E C C 4 4 5 5 6 6 10 10 11 11
Etoposide
 - + - + - + - + - + - + - +
300kDa
250kDa
180kDa
130kDa
100kDa
70kDa
50kDa
40kDa
ACTIN
