# Supplementary material for: A subset of CB002 xanthine analogs bypass p53-signaling to restore a p53 transcriptome and target an S-phase cell cycle checkpoint in tumors with mutated-p53
Source: eLife. 2021 Jul 29;10:e70429. doi: 10.7554/eLife.70429 (PMC8321552; doi:10.7554/eLife.70429)
Supplement: Source data 1. [file elife-70429-data1.zip › Blots/Hernandez Borrero et al whole western blots final.pdf]

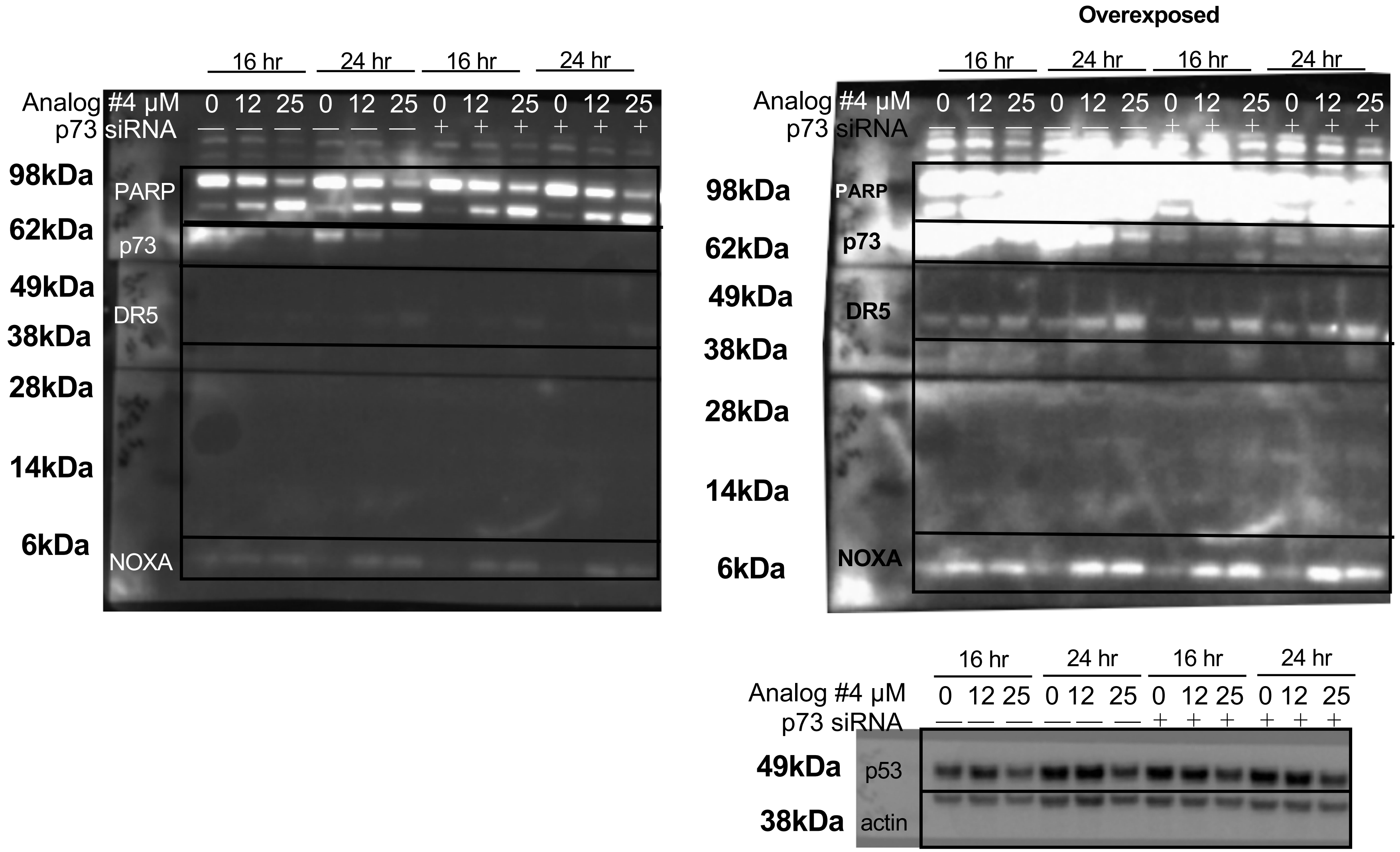
Overexposed
16 hr
24 hr
16 hr
24 hr
Analog #4 μM
0 12 25 0 12 25 0 12 25 0 12 25
p73 siRNA
— — — — — — + + + + + +
98kDa
62kDa
49kDa
38kDa
28kDa
14kDa
6kDa
PARP
p73
DR5
NOXA
p53
actin

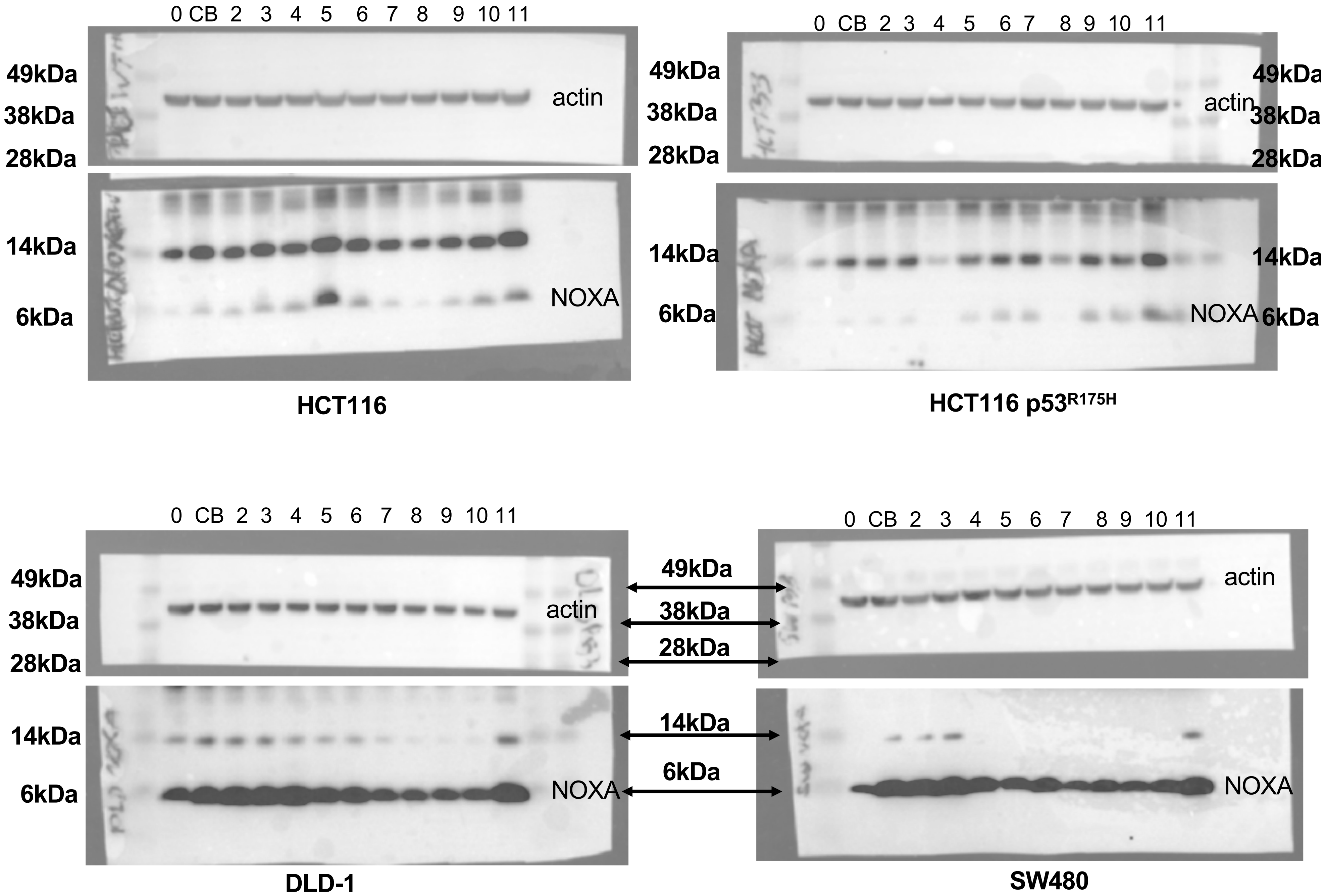
0 CB 2 3 4 5 6 7 8 9 10 11
49kDa
38kDa
28kDa
14kDa
6kDa
actin
NOXA
HCT116
0 CB 2 3 4 5 6 7 8 9 10 11
49kDa
38kDa
28kDa
14kDa
6kDa
actin
NOXA
HCT116 p53R175H
0 CB 2 3 4 5 6 7 8 9 10 11
49kDa
38kDa
28kDa
14kDa
6kDa
actin
NOXA
DLD-1
0 CB 2 3 4 5 6 7 8 9 10 11
actin
NOXA
SW480

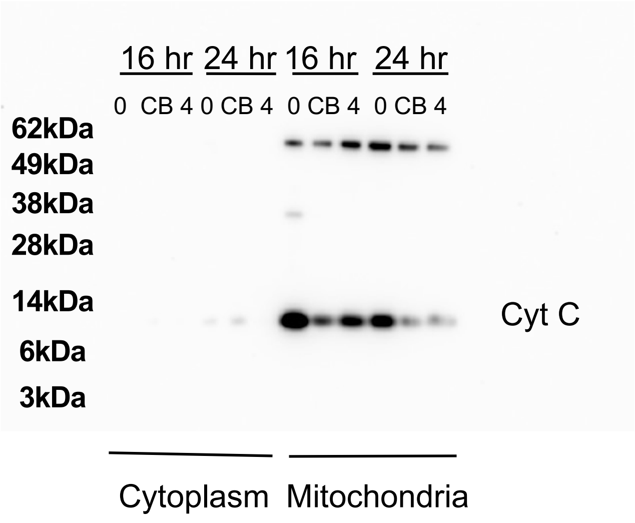
16 hr 24 hr 16 hr 24 hr
0 CB 4 0 CB 4 0 CB 4 0 CB 4
62kDa
49kDa
38kDa
28kDa
14kDa
6kDa
3kDa
Cyt C
Cytoplasm
Mitochondria

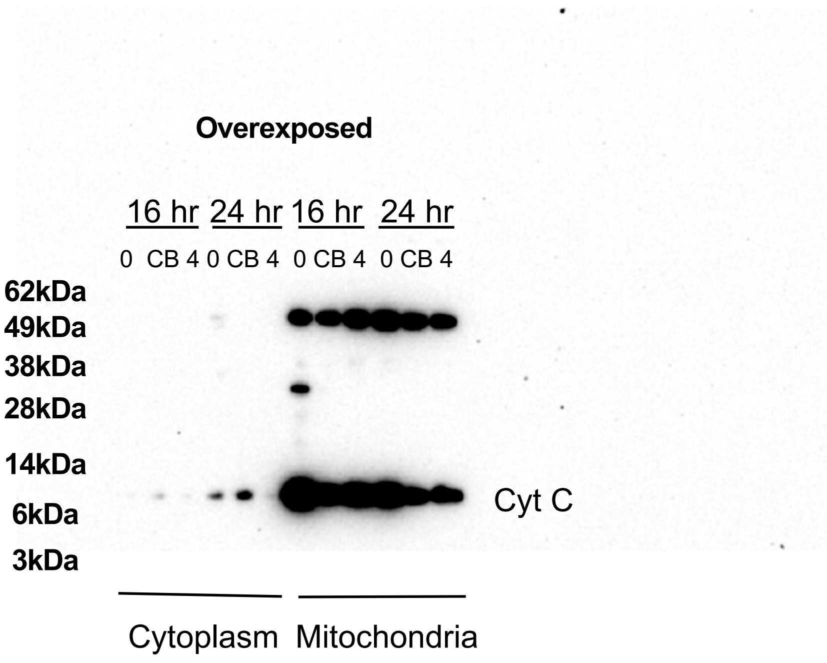
Overexposed
16 hr 24 hr 16 hr 24 hr
0 CB 4 0 CB 4 0 CB 4 0 CB 4
62kDa
49kDa
38kDa
28kDa
14kDa
6kDa
3kDa
Cyt C
Cytoplasm
Mitochondria

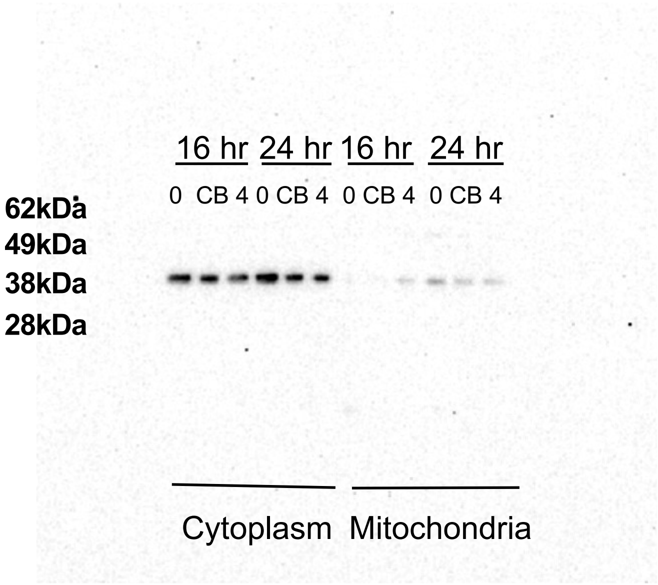
16 hr 24 hr 16 hr 24 hr
0 CB 4 0 CB 4 0 CB 4 0 CB 4
62kDa
49kDa
38kDa
28kDa
Cytoplasm
Mitochondria

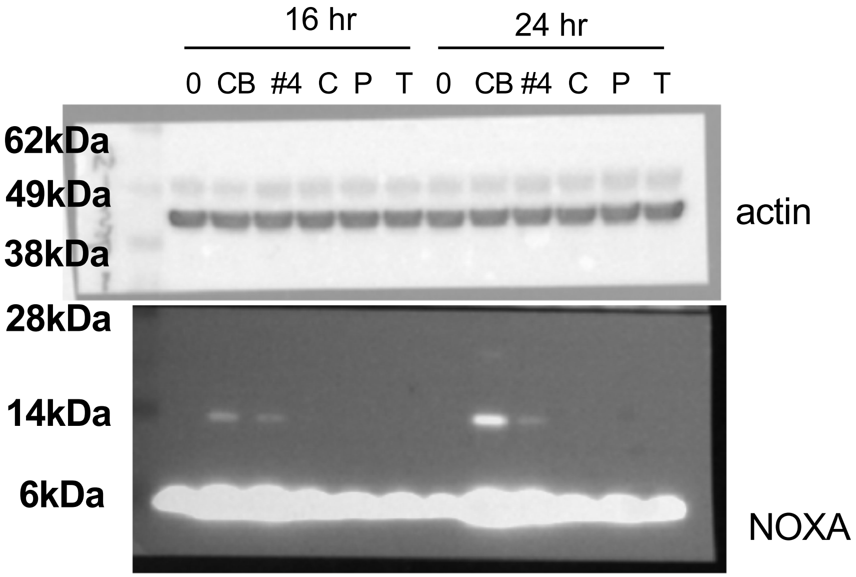
16 hr
24 hr
0 CB #4 C P T 0 CB #4 C P T
62kDa
49kDa
38kDa
28kDa
14kDa
6kDa
actin
NOXA

SW480

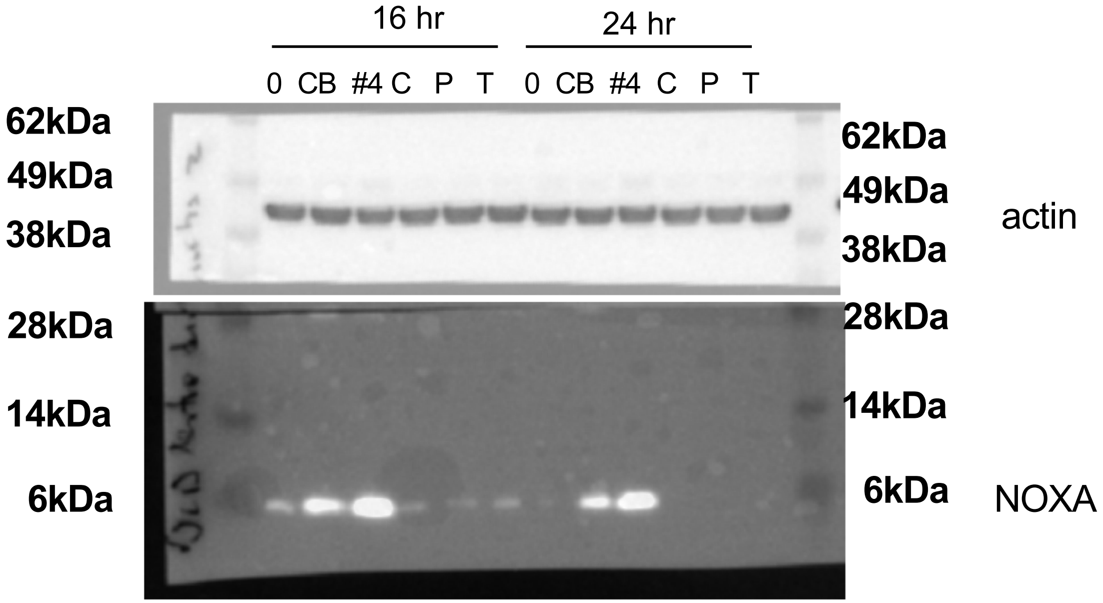
16 hr
24 hr
0 CB #4 C P T 0 CB #4 C P T
62kDa
49kDa
38kDa
28kDa
14kDa
6kDa
62kDa
49kDa
38kDa
28kDa
14kDa
6kDa
actin
NOXA

DLD-1

24 hr 48 hr
0 CB #4 0 CB #4 0 CB #4 0 CB #4
ATF3/4 siRNA — — — + + + — — — + + +

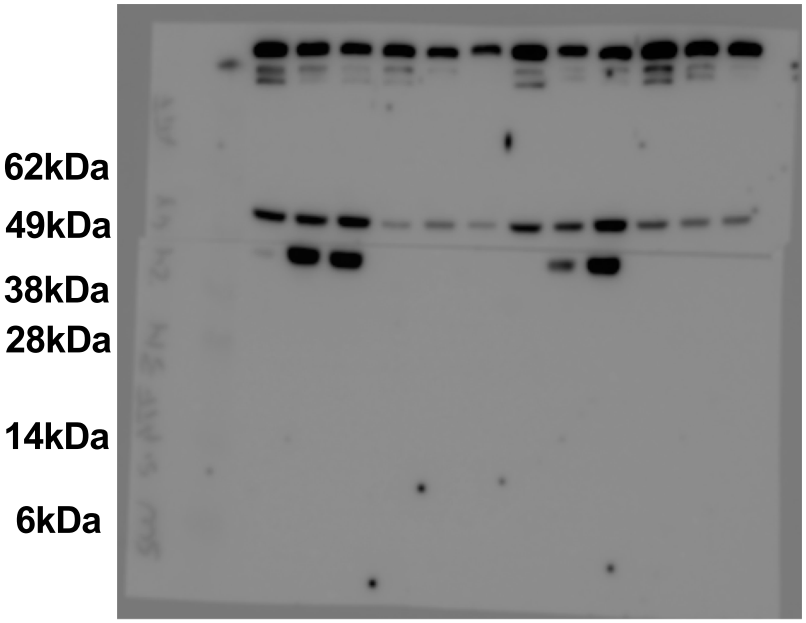

24 hr 48 hr
0 CB #4 0 CB #4 0 CB #4 0 CB #4
ATF3/4 siRNA — — — + + + — — — + + +

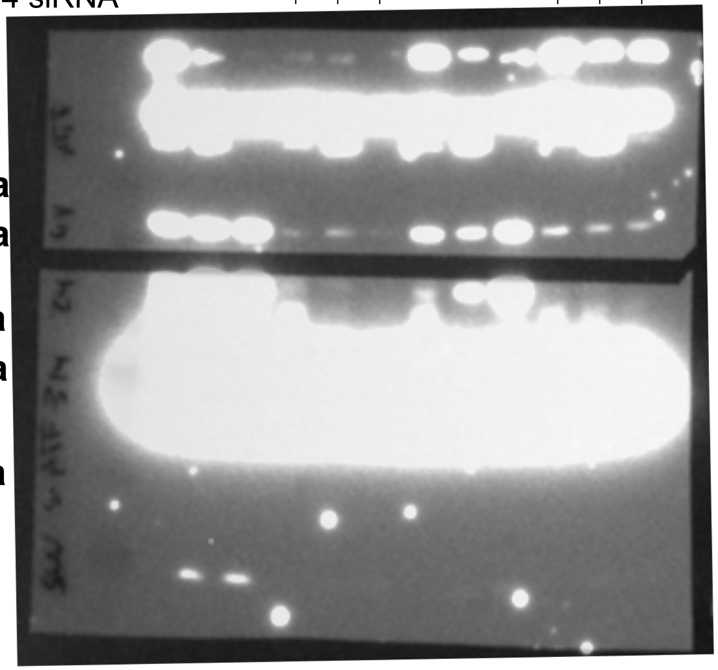

Overexposed

24 hr 48 hr
0 CB #4 0 CB #4 0 CB #4 0 CB #4
ATF3/4 siRNA — — — + + + — — — + + +

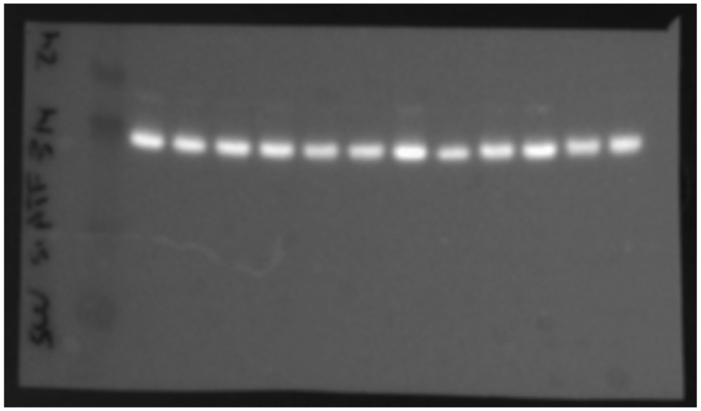

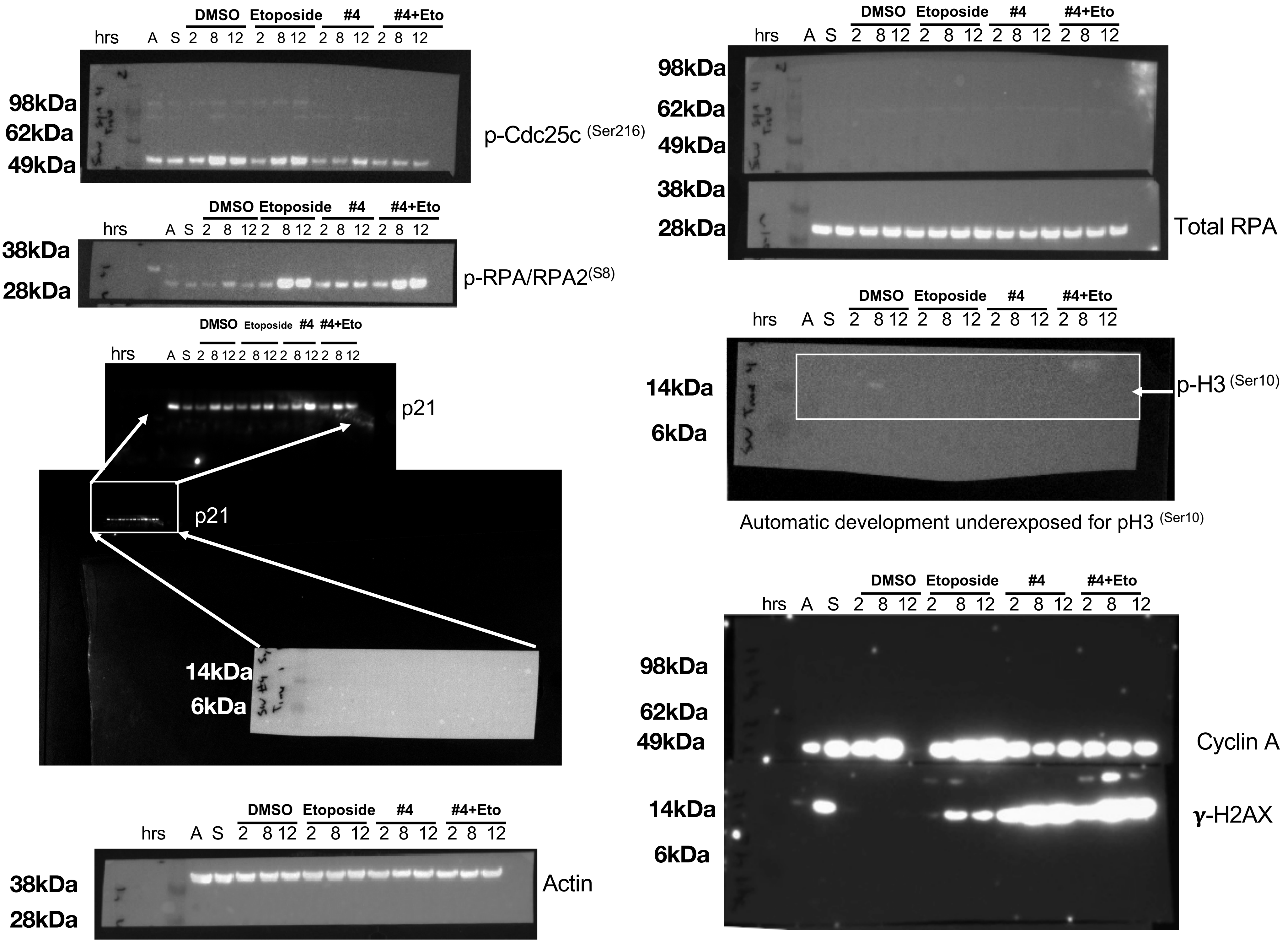
DMSO Etoposide #4 #4+Eto
hrs A S 2 8 12 2 8 12 2 8 12 2 8 12
98kDa
62kDa
49kDa
p-Cdc25c (Ser216)
38kDa
28kDa
p-RPA/RPA2(S8)
p21
p21
14kDa
6kDa
Actin
Total RPA
p-H3 (Ser10)
Automatic development underexposed for pH3 (Ser10)
Cyclin A
γ-H2AX

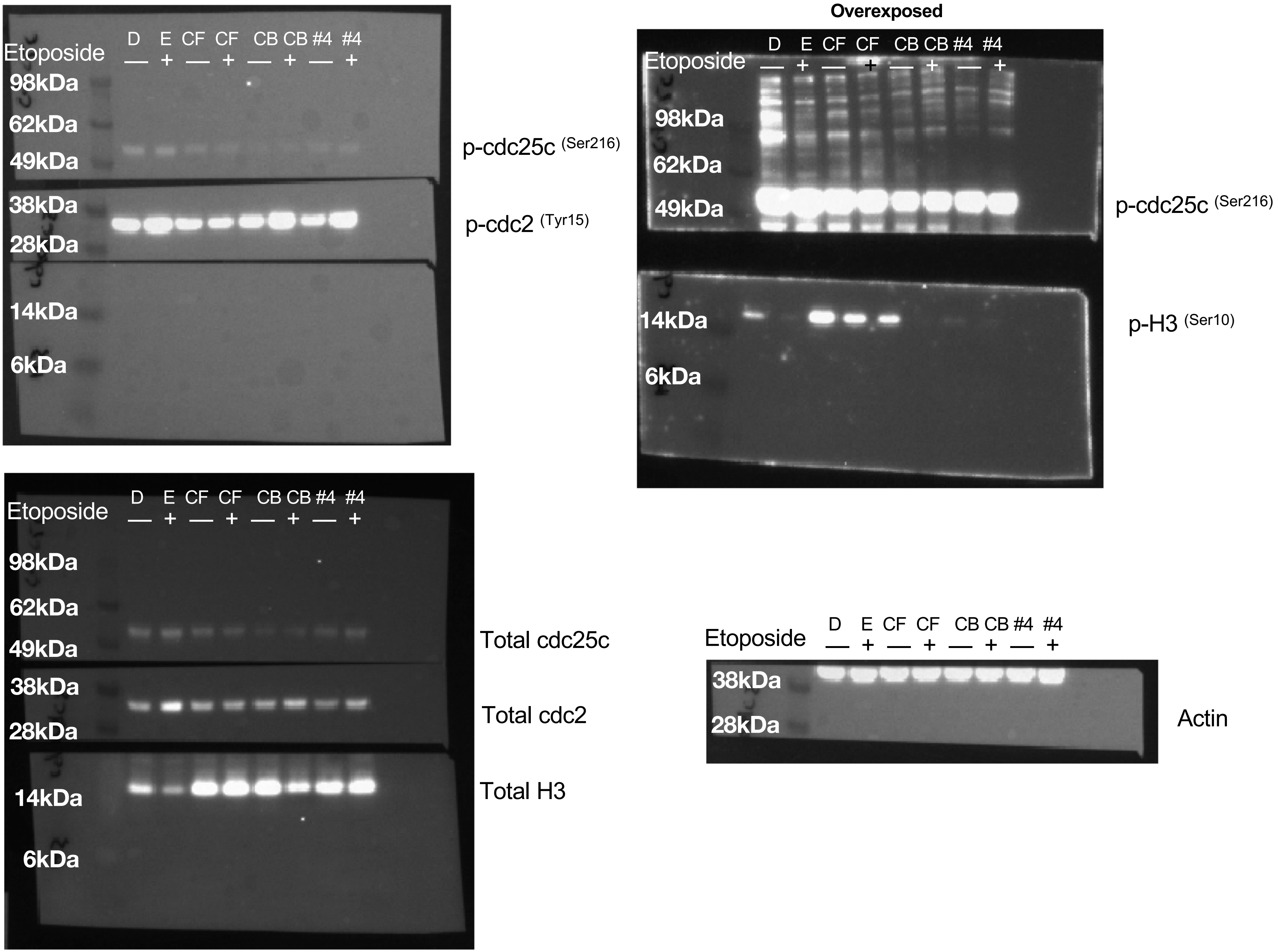

Overexposed
Etoposide
D E CF CF CB CB #4 #4
— + — + — + — +
98kDa
62kDa
49kDa
38kDa
28kDa
14kDa
6kDa
p-cdc25c (Ser216)
p-cdc2 (Tyr15)
p-H3 (Ser10)
Total cdc25c
Total cdc2
Total H3
Actin

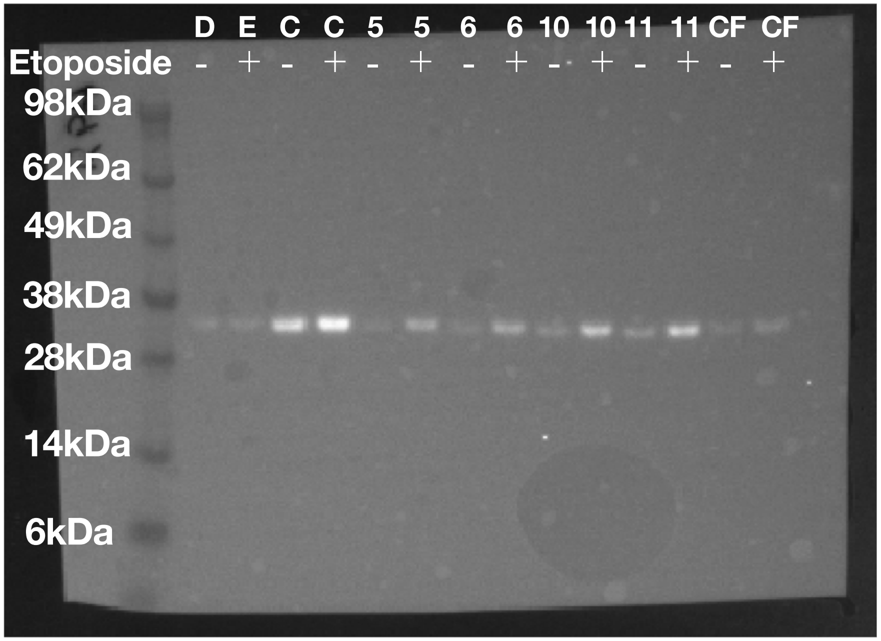

p-RPA/RPA2(S8)

Overexposed

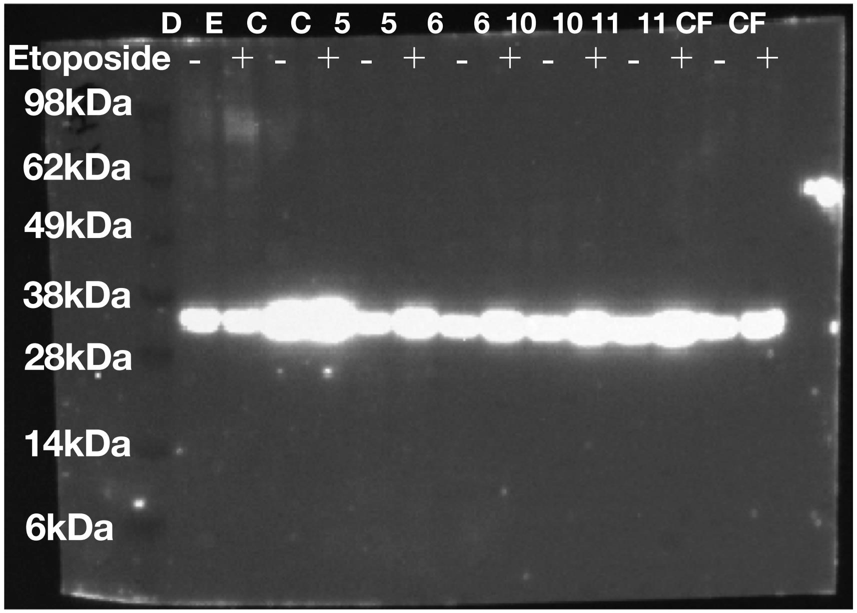

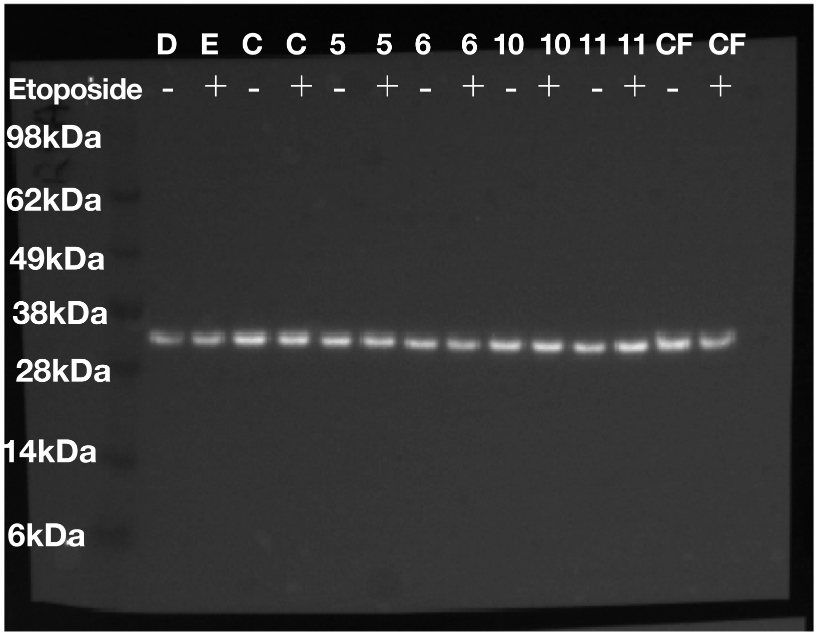

Total RPA/RPA2

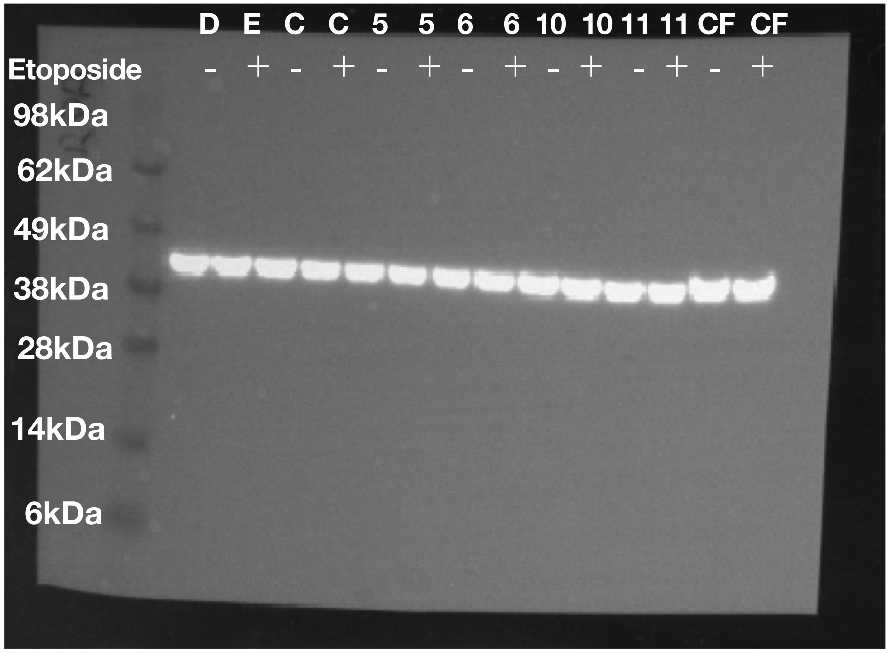

ACTIN

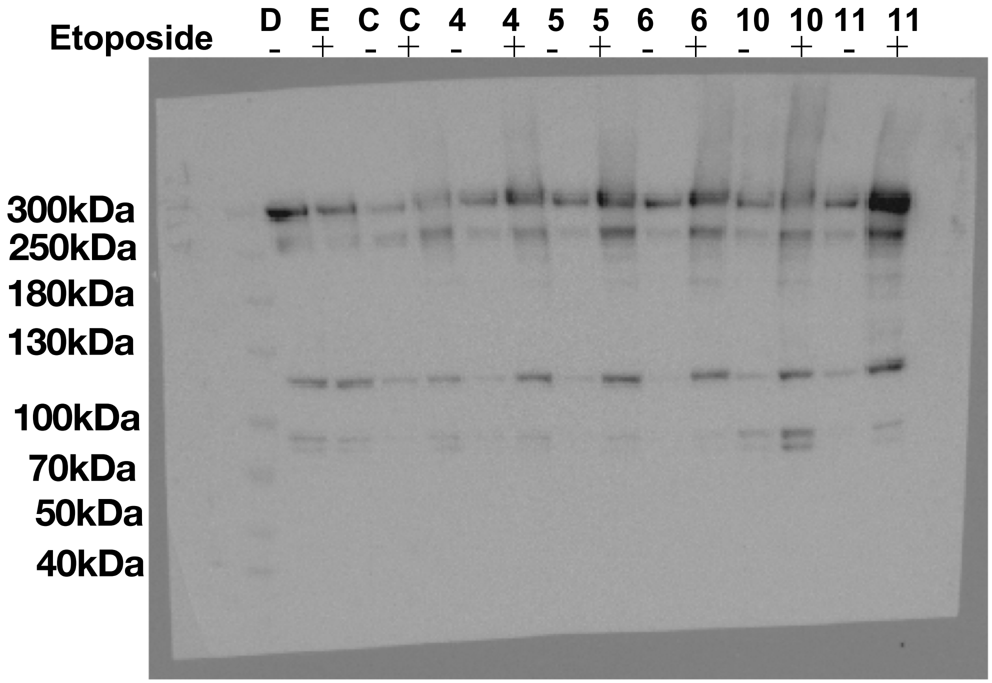

p-ATR$^{(Thr1989)}$

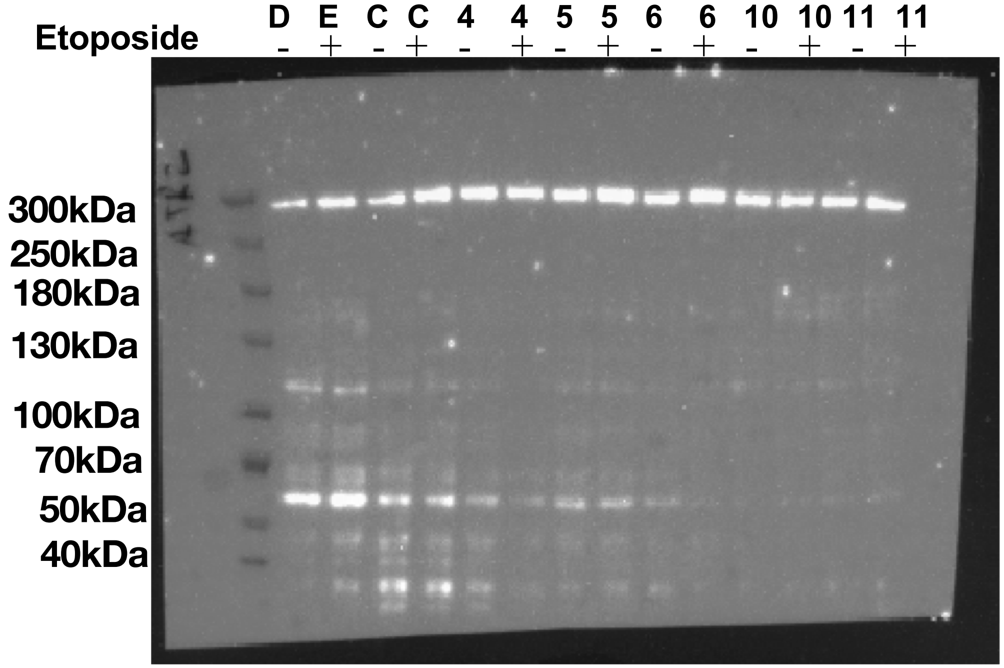

Total ATR

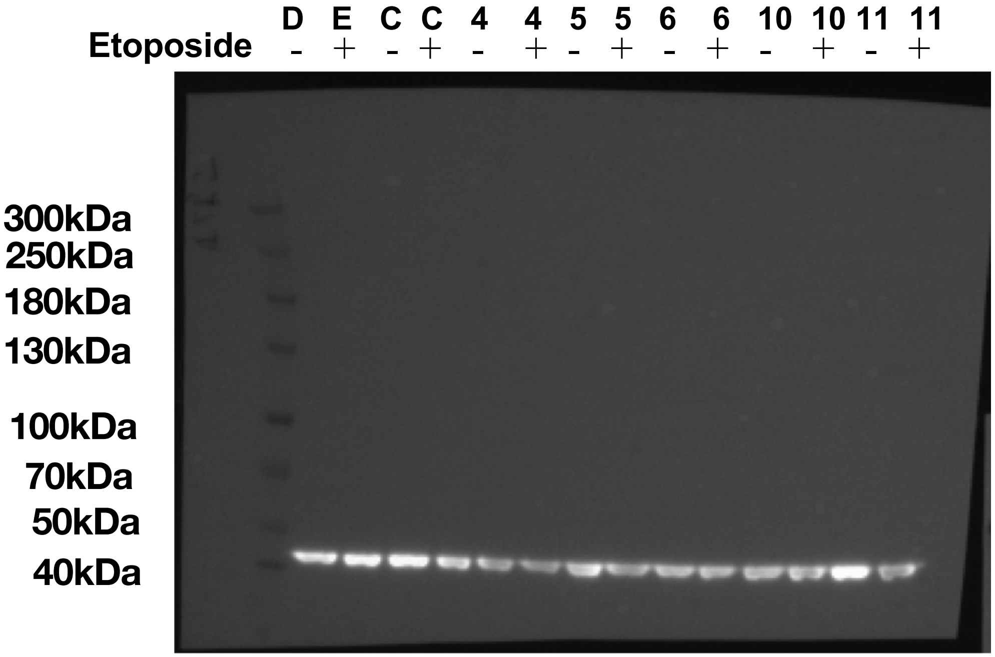

ACTIN
