# Supplementary figures and images for: A subset of CB002 xanthine analogs bypass p53-signaling to restore a p53 transcriptome and target an S-phase cell cycle checkpoint in tumors with mutated-p53
Source: eLife. 2021 Jul 29;10:e70429. doi: 10.7554/eLife.70429 (PMC8321552; doi:10.7554/eLife.70429)

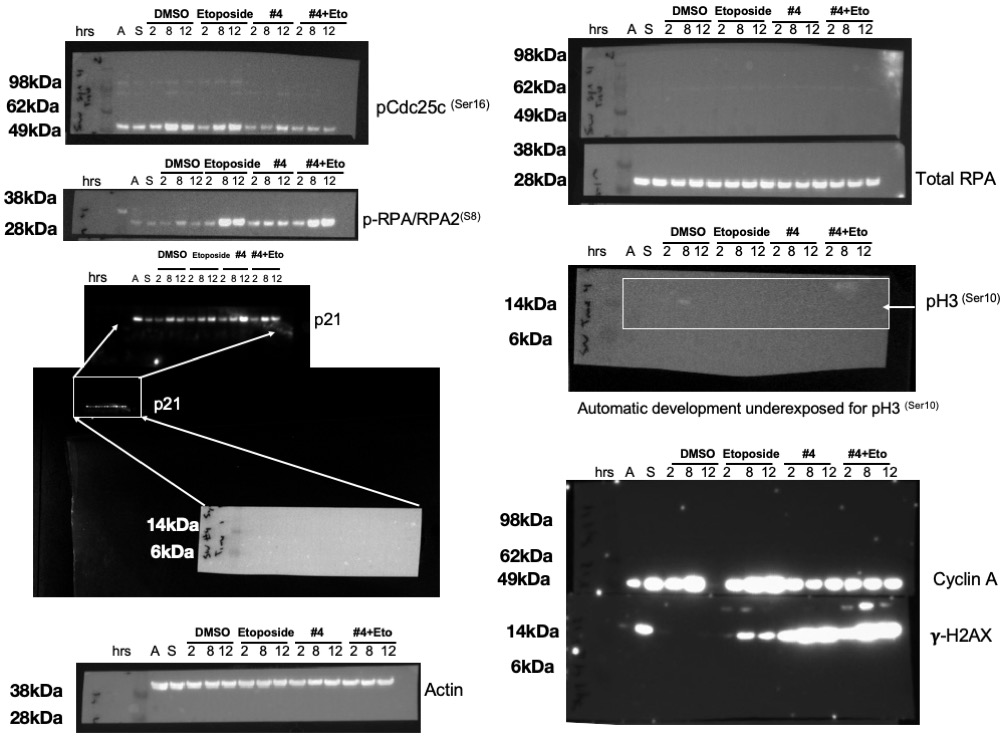

Supplement: Source data 1. [file elife-70429-data1.zip › Blots/Source Data Figure 5D.jpeg]

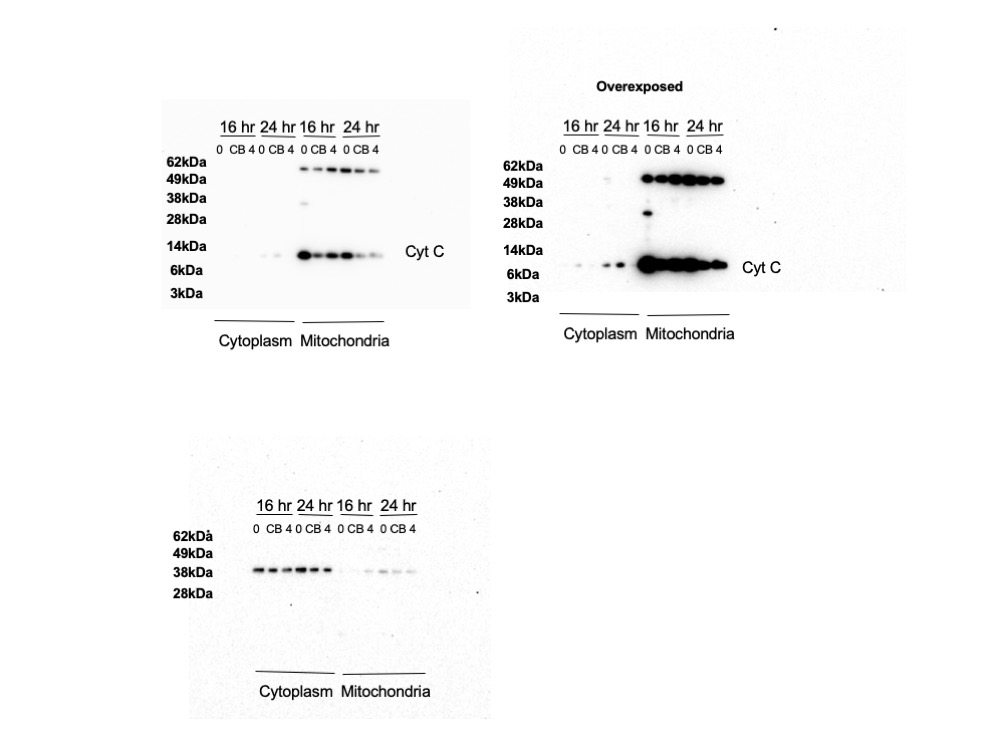

Supplement: Source data 1. [file elife-70429-data1.zip › Blots/Source Data Figure 1E.jpeg]

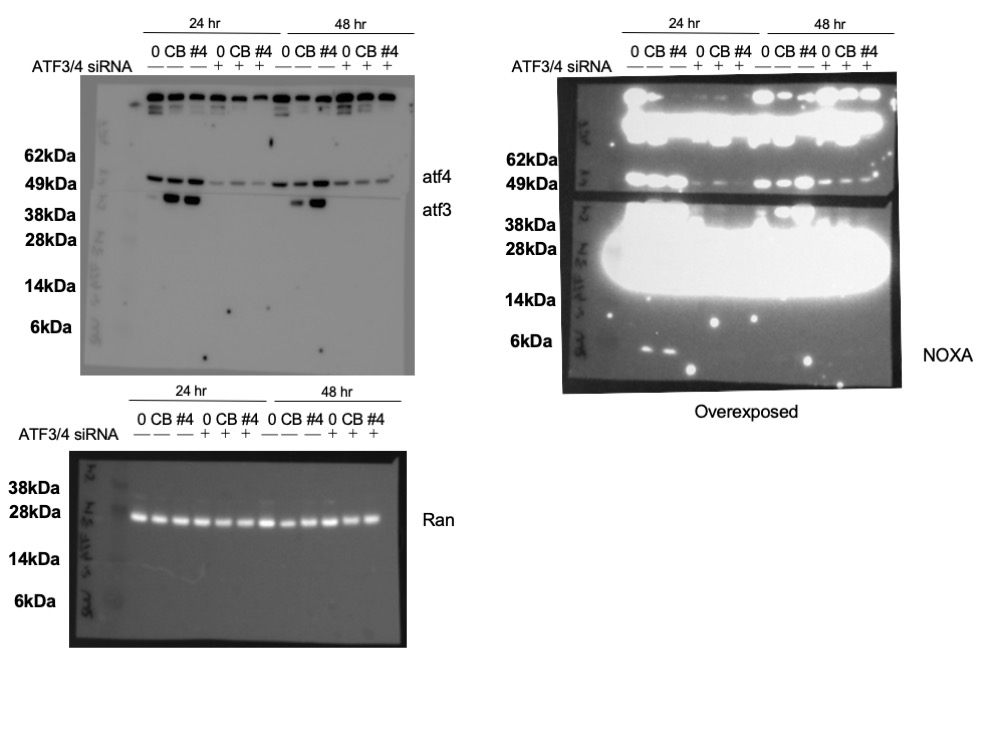

Supplement: Source data 1. [file elife-70429-data1.zip › Blots/Source Data Figure 1I.jpeg]

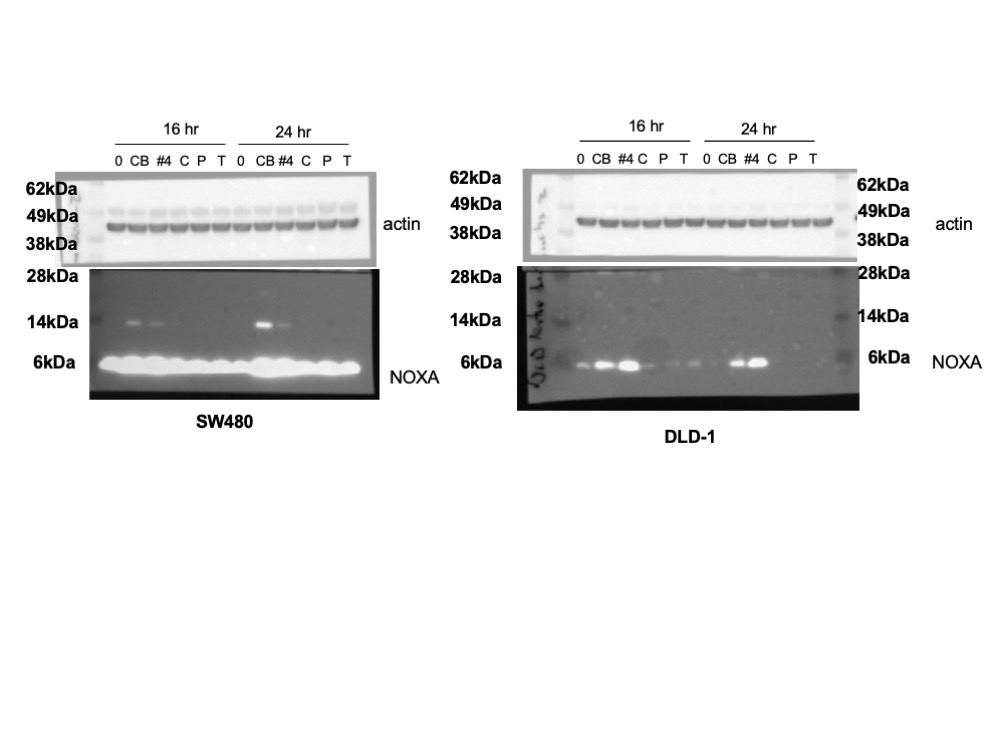

Supplement: Source data 1. [file elife-70429-data1.zip › Blots/Source Data Figure 1H.jpeg]

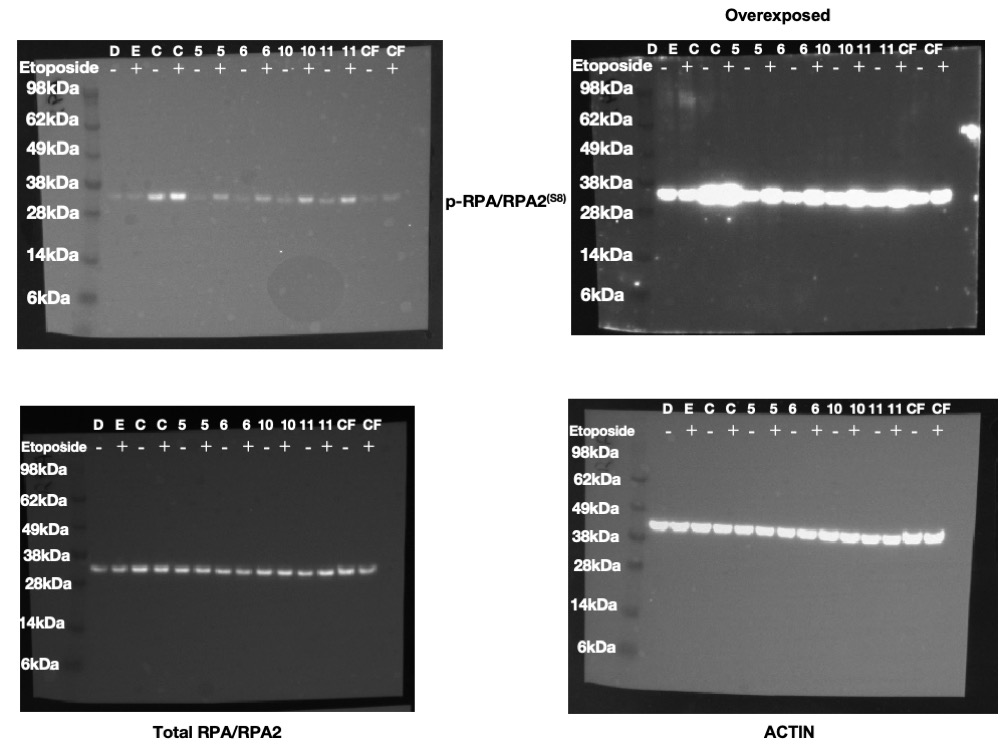

Supplement: Source data 1. [file elife-70429-data1.zip › Blots/Source Data Figure 5B.jpeg]

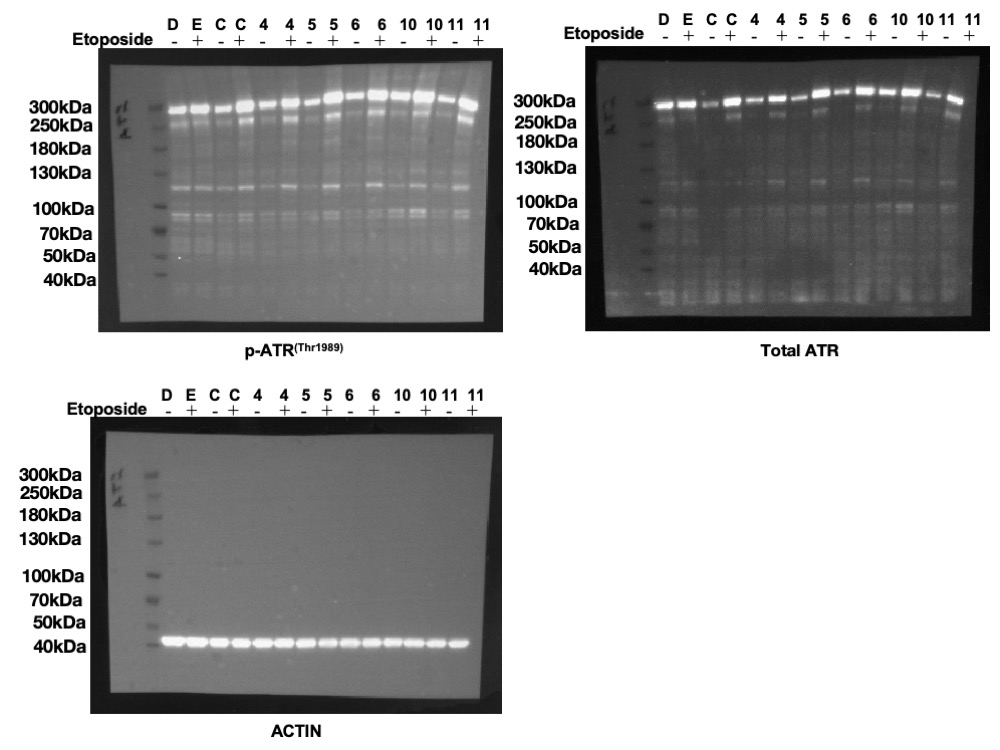

Supplement: Source data 1. [file elife-70429-data1.zip › Blots/Source Data Figure 5C.jpeg]

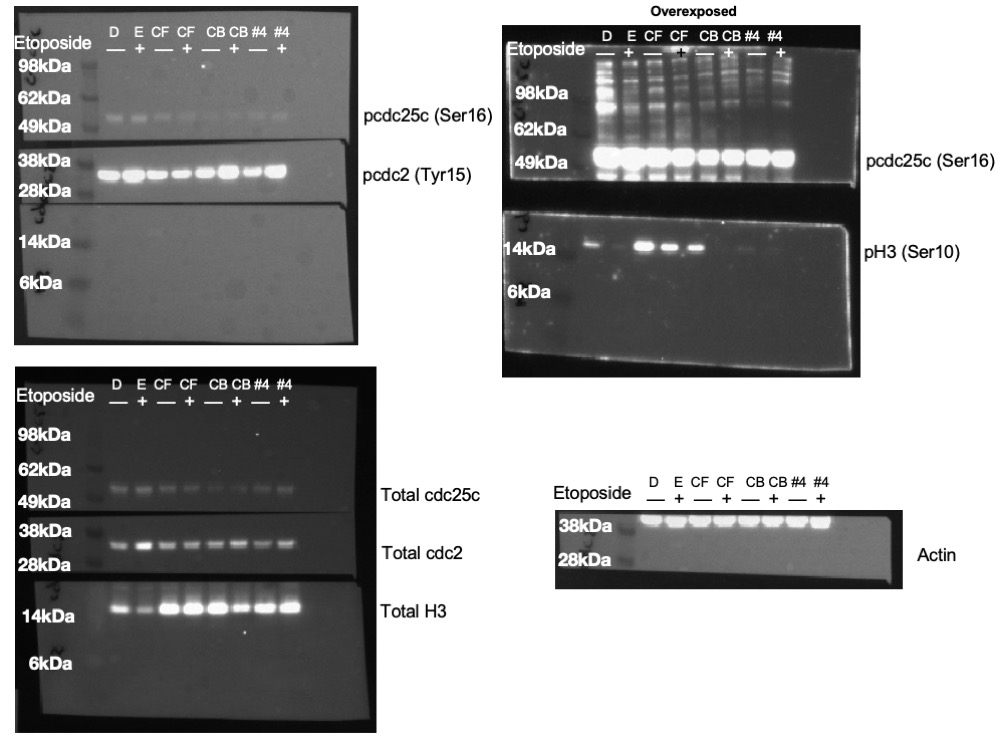

Supplement: Source data 1. [file elife-70429-data1.zip › Blots/Source Data Figure 5A.jpeg]

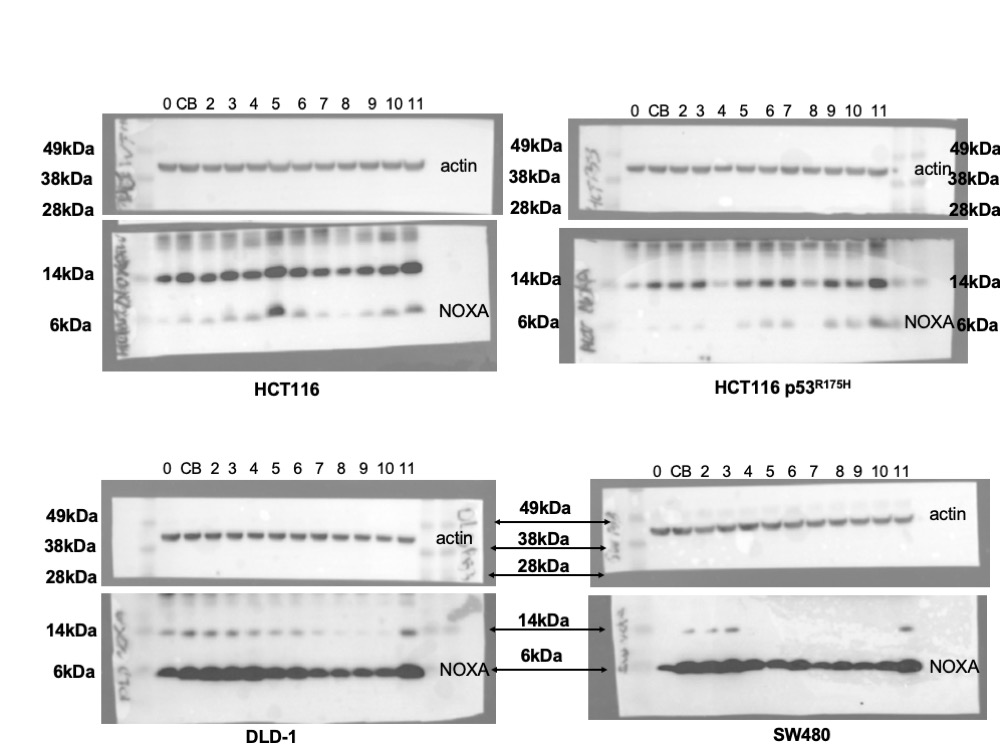

Supplement: Source data 1. [file elife-70429-data1.zip › Blots/Source Data Figure 1G.jpeg]

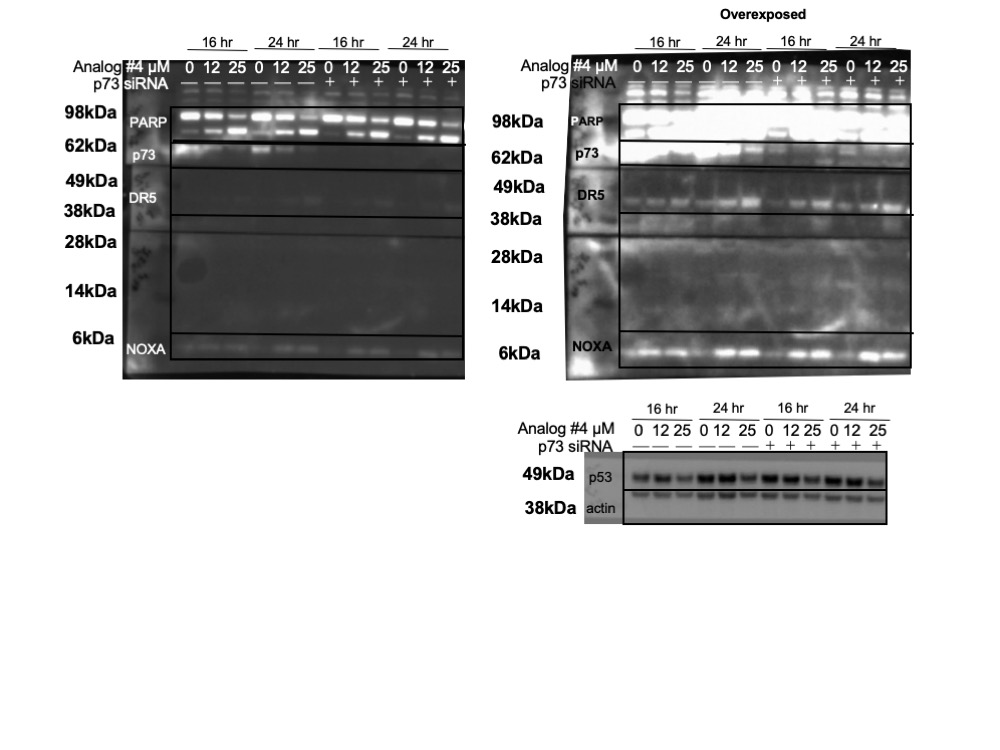

Supplement: Source data 1. [file elife-70429-data1.zip › Blots/Source Data Figure 1F.jpeg]
