# Supplementary material for: A subset of CB002 xanthine analogs bypass p53-signaling to restore a p53 transcriptome and target an S-phase cell cycle checkpoint in tumors with mutated-p53
Source: eLife. 2021 Jul 29;10:e70429. doi: 10.7554/eLife.70429 (PMC8321552; doi:10.7554/eLife.70429)
Supplement: Transparent reporting form [file elife-70429-transrepform.pdf]

Westbrook Centre
Milton Road
Cambridge CB4 1YG, UK

P 01223 855340
W elifesciences.org
T @elife

eLife's transparent reporting form

We encourage authors to provide detailed information *within their submission* to facilitate the interpretation and replication of experiments. Authors can upload supporting documentation to indicate the use of appropriate reporting guidelines for health-related research (see EQUATOR Network), life science research (see the BioSharing Information Resource), or the ARRIVE guidelines for reporting work involving animal research. Where applicable, authors should refer to any relevant reporting standards documents in this form.

If you have any questions, please consult our Journal Policies and/or contact us: editorial@elifesciences.org.

Sample-size estimation

- You should state whether an appropriate sample size was computed when the study was being designed
- You should state the statistical method of sample size computation and any required assumptions
- If no explicit power analysis was used, you should describe how you decided what sample (replicate) size (number) to use

Please outline where this information can be found within the submission (e.g., sections or figure legends), or explain why this information doesn't apply to your submission:

In vivo experiment sample size was determined as n=10 mice per cohort. Tumor volume experiments were compared at a fixed time point between analogue #4 treatment and DMSO vehicle controls. Sample size calculation was determined as follows using the approximation for tumor volume and solving the equations in order to achieve statistical power between treatment and control:

z(0.8)*sqrt[(s(c)^2+ s(e)^2)/n] – z(0.06)*s(c)*sqrt(2/n) = mu(c) –mu(e)

where z(0.8) = 0.842, z(0.06) = -1.555 are the quantities associated with 80% power and 6% type I error, n = number animals per group, mu(e) is the postulated mean volume among experimental group and mu(c) is that of the control animals, and s(c) and s(e) are standard deviations of control volumes and experimental volumes, respectively. Substituting n = 10, mu(e) = mu(c)/2 results in the requirement that:
z(0.8)*sqrt[(s(c)^2+ s(e)^2)/n] – z(0.06)*s(c)*sqrt(2/n) be less than or equal to mu(c) –mu(e). Tumor volumes are bounded below by zero so we approximated the two standard deviations by s(c) = mu(c)/2 and s(e) = mu(e)/2. Making these substitutions plus setting mu(e) = mu(c)/2 results in the requirement that z(.8)*sqrt(1.25/n)-z(0.06)*sqrt(2/n) be less than or equal to 1. With n=10 the above quantity equals 0.9929 which justifies that 10 animals per group result in 80% power and 6% type I error.

Experimental information can be found in the materials and methods section as well as the Figure 6 legend.

eLife Sciences Publications, Ltd is a limited liability non-profit non-stock corporation incorporated in the State of Delaware, USA, with company number 5030732, and is registered in the UK with company number FC030576 and branch number BR015634 at the address Westbrook Centre, Milton Road, Cambridge CB4 1YG.

Replicates

- You should report how often each experiment was performed
- You should include a definition of biological versus technical replication
- The data obtained should be provided and sufficient information should be provided to indicate the number of independent biological and/or technical replicates
- If you encountered any outliers, you should describe how these were handled
- Criteria for exclusion/inclusion of data should be clearly stated
- High-throughput sequence data should be uploaded before submission, with a private link for reviewers provided (these are available from both GEO and ArrayExpress)

Please outline where this information can be found within the submission (e.g., sections or figure legends), or explain why this information doesn't apply to your submission:

The number of biological replicates can be found in the materials and methods section, Figure legends or supplemental files description when applicable.

eLife Sciences Publications, Ltd is a limited liability non-profit non-stock corporation incorporated in the State of Delaware, USA, with company number 5030732, and is registered in the UK with company number FC030576 and branch number BR015634 at the address Westbrook Centre, Milton Road, Cambridge CB4 1YG.

Statistical reporting

- Statistical analysis methods should be described and justified
- Raw data should be presented in figures whenever informative to do so (typically when N per group is less than 10)
- For each experiment, you should identify the statistical tests used, exact values of N, definitions of center, methods of multiple test correction, and dispersion and precision measures (e.g., mean, median, SD, SEM, confidence intervals; and, for the major substantive results, a measure of effect size (e.g., Pearson's r, Cohen's d)
- Report exact p-values wherever possible alongside the summary statistics and 95% confidence intervals. These should be reported for all key questions and not only when the p-value is less than 0.05.

Please outline where this information can be found within the submission (e.g., sections or figure legends), or explain why this information doesn't apply to your submission:

Statistical information of applicable experiments can be found in the materials and methods section as well as in the Figure legends.

(For large datasets, or papers with a very large number of statistical tests, you may upload a single table file with tests, Ns, etc., with reference to sections in the manuscript.)

Group allocation

- Indicate how samples were allocated into experimental groups (in the case of clinical studies, please specify allocation to treatment method); if randomization was used, please also state if restricted randomization was applied
- Indicate if masking was used during group allocation, data collection and/or data analysis

Please outline where this information can be found within the submission (e.g., sections or figure legends), or explain why this information doesn't apply to your submission:

No clinical studies were performed in the presented manuscript therefore group allocation does not apply to the submission.

eLife Sciences Publications, Ltd is a limited liability non-profit non-stock corporation incorporated in the State of Delaware, USA, with company number 5030732, and is registered in the UK with company number FC030576 and branch number BR015634 at the address Westbrook Centre, Milton Road, Cambridge CB4 1YG.

Additional data files ("source data")

- We encourage you to upload relevant additional data files, such as numerical data that are represented as a graph in a figure, or as a summary table
- Where provided, these should be in the most useful format, and they can be uploaded as "Source data" files linked to a main figure or table
- Include model definition files including the full list of parameters used
- Include code used for data analysis (e.g., R, MatLab)
- Avoid stating that data files are "available upon request"

Please indicate the figures or tables for which source data files have been provided:

> Additional data files are in a zip file as source data with respective relation to main figure. Data analysis information can be found in the materials and method section. For microarray and proteomic analysis, raw data was supplied as supplemental tables with table descriptions.

eLife Sciences Publications, Ltd is a limited liability non-profit non-stock corporation incorporated in the State of Delaware, USA, with company number 5030732, and is registered in the UK with company number FC030576 and branch number BR015634 at the address Westbrook Centre, Milton Road, Cambridge CB4 1YG.
